# A computationally efficient biomedical text processing framework for pharmacovigilance: integrating low-rank adaptation and interpretable AI for adverse drug reaction detection

**DOI:** 10.1007/s11517-025-03477-w

**Published:** 2025-11-29

**Authors:** Zahra Rezaei, Sara Safi Samghabadi, Mohammad Amin Amini, Yaser Mike Banad

**Affiliations:** School of Electrical and Computer Engineering, University of Oklahoma, Norman, OK 73019 USA

**Keywords:** Adverse drug reaction, Low-rank adaptation (LoRA), QLoRA, Encoder-only transformers, Shapley additive explanations (SHAP), Computational pharmacovigilance, Healthcare information systems

## Abstract

Early detection of adverse drug reactions (ADRs) is crucial for patient safety but remains challenging due to underreporting and delayed data in traditional pharmacovigilance. This study proposes a computationally efficient and interpretable framework for ADR detection by integrating Low-Rank Adaptation (LoRA) and SHapley Additive Explanations (SHAP) with encoder-based transformer models (BERT, DistilBERT, RoBERTa). Leveraging over 3,900 annotated tweets, our approach demonstrates that LoRA reduces trainable parameters and training costs by up to 50%, while preserving high classification accuracy (above 98%) across three disease classes. SHAP analysis provides actionable interpretability, revealing that the models consistently rely on clinically relevant terms, such as drug names and symptoms, to drive predictions. Compared to traditional finetuning, LoRA and Efficient Finetuning of Quantized LLMs (QLoRA) offer a robust and scalable alternative for processing noisy, informal social media data, making real-time ADR monitoring feasible in resource-constrained healthcare settings. This framework strikes a balance between computational efficiency, interpretability, and predictive performance, supporting the integration of pharmacovigilance into clinical decision support systems for safer patient care.

**Graphical Abstract:**

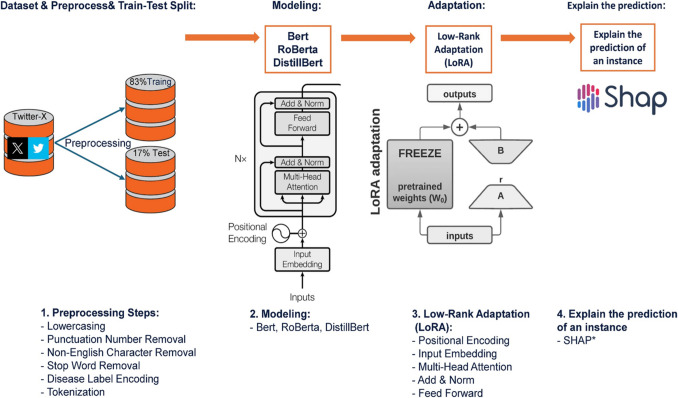

## Introduction

Effective pharmacovigilance constitutes a critical component of healthcare infrastructure, directly impacting patient safety, clinical decision-making, and biomedical research. Within this domain, computational biological engineering, particularly through the development of advanced algorithms for biomedical text analysis and clinical decision support, is essential for improving the effectiveness of Adverse Drug Reaction (ADR) detection systems. The integration of real-time ADR detection capabilities into healthcare information systems can significantly enhance clinician responsiveness, optimize drug safety protocols, and foster biomedical data interoperability across diverse healthcare settings. Within the domain of medicine, pharmacovigilance (PV) is dedicated to helping physicians ‘do not harm’. PV requires monitoring the effects of pharmaceutical products after licensure and includes ongoing surveillance of known side effects of medicines, as well as sifting through large volumes of data to identify and act on emerging, previously unknown side effects. For this reason, AI/ML appears to be well-suited to perform PV tasks, given the large volume of data, high degree of uncertainty, and the need to learn from data [1]. The integration of ADR detection systems into existing healthcare information technologies represents a critical advancement in patient safety monitoring. Current healthcare information systems often lack efficient mechanisms for incorporating real-time patient-reported experiences, resulting in a noticeable gap between clinical observations and patients’ actual experiences. Our proposed framework aims to address this deficiency by offering a computationally efficient methodology readily integrable into existing healthcare information infrastructures. From a biomedical engineering standpoint, ADR detection poses a complex signal processing challenge: accurately extracting the ‘signal’ (genuine ADR mentions) from the ‘noise’ (unrelated social media content). Our approach leverages advanced transformer architectures to process these linguistic signals with high precision while maintaining clinical interpretability. The global escalation of chronic diseases further amplifies this challenge, with millions worldwide relying on long-term pharmacotherapy for conditions such as diabetes, cardiovascular diseases, and cancer. In the United States alone, over 140 million individuals live with chronic illnesses, underscoring the urgent demand for safe and effective therapeutic interventions [2]. However, while these medications are essential for disease management, they may also lead to ADRs, unintended and sometimes severe side effects. ADRs can range from mild discomfort to life-threatening complications, making early detection and monitoring critical for patient safety. Healthcare professionals, regulatory agencies, and pharmaceutical companies rely on ADR data to guide drug development, prescribing decisions, and post-marketing surveillance. Traditional PV systems, though foundational, often grapple with inherent limitations in their ability to comprehensively and efficiently monitor ADRs. These methods are typically resource-intensive, constrained by human processing capacity. They can result in significant delays in detecting critical safety signals from the vast and ever-growing volume of diverse data sources. This underscores a crucial gap between the sheer scale of available information and the timely identification of emergent drug safety concerns, highlighting the urgent need for more advanced and scalable solutions to enhance drug safety and patient outcomes [3].

Conventional pharmacovigilance methods for detecting ADRs traditionally encompass clinical trials, spontaneous reporting systems (e.g., the FDA’s Adverse Event Reporting System—FAERS), and extensive literature-based mining. However, these established approaches are frequently hampered by several critical limitations that impede comprehensive drug safety surveillance [4]. A significant challenge in pharmacovigilance is the ongoing problem of underreporting and delayed detection. This issue arises mainly because systems like the FDA Adverse Event Reporting System (FAERS) heavily depend on voluntary submissions, which lead to significant data gaps and reporting biases. Additionally, the limited scope of clinical trials, often characterized by tightly controlled environments and small sample sizes, significantly restricts their ability to generalize findings. As a result, these trials are usually inadequate in identifying rare or long-term adverse effects [5]. Finally, the manual analysis of scientific publications for ADR detection is essential but remains a time-consuming, resource-intensive, and inherently challenging process to scale effectively [6].

With the rise of social media platforms, particularly X (formerly Twitter), a new source of real-time patient-reported adverse drug reactions (ADRs) has emerged. These platforms provide vast amounts of user-generated content, enabling immediate access to patient experiences with medications [7]. Unlike traditional reporting systems, social media facilitates earlier detection of emerging drug safety concerns, enhancing established pharmacovigilance efforts. However, analyzing unstructured, noisy social media data poses significant challenges, including informal language, spelling variations, slang, and contextual ambiguity [8]. Leveraging NLP and Transformer-Based Models for ADR Detection: Recent advancements in Natural Language Processing (NLP) and Large Language Models (LLMs) have significantly enhanced automated text analysis in ADR detection. Transformer-based models, such as BERT, DistilBERT, and RoBERTa, have shown exceptional capabilities in understanding linguistic patterns and contextual relationships, making them highly effective for processing ADR-related social media data [9].

Numerous studies have explored the potential of deep learning in ADR detection:

- Predicting Drug Withdrawals: Transformer-based models have demonstrated the ability to predict drug market withdrawals, achieving AUC scores above 0.75 [10].Medication Change Classification: BERT-based models outperform traditional NLP methods in detecting medication changes in patient records [11]. Deep Learning for ADR Identification: Some studies have employed TextCNN models and hybrid deep learning-topic modeling approaches to extract ADRs from social media [12]. Uncovering Drug-Side Effect Associations: Researchers have developed integrated approaches combining deep learning, topic modeling, and classification to identify and label ADRs in temporal text corpora, addressing challenges such as data structure, class imbalance, and language variations in social media [13]. A descriptive analysis of adverse events (AEs) for newly introduced multiple myeloma (MM) medications, leveraging data from the FDA Adverse Event Reporting System (FAERS) spanning 2015 to 2022, indicated that ixazomib was associated with the highest volume of reported AEs, including fatalities, in comparison to other novel MM treatments. This study highlights the crucial importance of vigilant surveillance for potential safety signals associated with emerging MM therapies, particularly among younger patients [14]. Further advancing the capabilities in biomedical text analysis, advanced deep learning models, particularly Convolutional Neural Networks (CNNs), Bidirectional Encoder Representations from Transformers (BERT), and Generative Pre-trained Transformers (GPT), have emerged as powerful tools for identifying complex patterns within clinical trial data that conventional methods might overlook. The integration of Natural Language Processing (NLP) techniques, in particular, enables the practical analysis of unstructured data, such as clinical notes and patient reports, providing a more comprehensive understanding of ADRs. This multi-faceted approach aims to significantly improve the accuracy and efficiency of ADR detection, aligning with the fundamental principles of Good Clinical Practice (GCP) and ultimately enhancing pharmacovigilance practices [15]. BERT & RoBERTa for ADR Extraction: Fine-tuned BERT and RoBERTa models have significantly improved ADR classification accuracy, outperforming prior state-of-the-art models [2, 16]. Addressing Social Media Data Challenges: RoBERTa models fine-tuned on Twitter data have been shown to effectively handle linguistic diversity, data sparsity, and noise, significantly improving ADR mention classification [17]. DeBERTa for Automated ADR Reporting: A two-stage DeBERTa-based NLP algorithm trained on clinical discharge summaries has demonstrated superior performance in ADR detection, offering a new approach for pharmacovigilance [16]. ChatGPT for Pharmacovigilance: Recent studies have evaluated ChatGPT’s ability to detect ADRs, achieving 94.4% accuracy for general ADR detection and 99.3% accuracy for severe ADRs, indicating its potential for automated content analysis in biomedical research [18]. As AI models become increasingly sophisticated and accurate in their predictions, the need for model interpretability and explainability becomes increasingly critical. Without a clear understanding of how and why a prediction is formed, particularly in sensitive domains like pharmacovigilance, achieving trust and widespread acceptance of AI applications remains a significant challenge. Explainable AI (XAI) directly addresses this fundamental limitation of complex machine learning and deep learning algorithms, moving beyond their ‘black-box’ nature to provide human-comprehensible insights into their decision-making processes [19]. XAI for Drug-Drug Interactions Prediction: Recent research has explored the role of XAI in pharmacovigilance, introducing attention mechanisms and gradient-based methods to improve trust and reliability in drug interaction predictions [20]. ADE Extraction from Clinical Text: A systematic review highlighted various approaches, including rule-based, machine learning, deep learning, hybrid, and LLM-based methods, showcasing the importance of Named Entity Recognition (NER) and Relation Extraction (RE) techniques in ADR research [21].

Although the present study focuses exclusively on ADR-related tweets, this emphasis is intentional. Social media platforms,particularly Twitte, r represent one of the most dynamic and noise-prone environments for pharmacovigilance signal detection. Modeling ADR patterns under such conditions provides a rigorous test of model robustness, linguistic adaptability, and interpretability. The objective of this study is therefore not to replace formal biomedical systems such as electronic health records (EHRs) or clinical notes, but to establish a computationally efficient and interpretable foundation that can later be adapted to structured biomedical domains. By demonstrating effectiveness on unstructured, user-generated text, the proposed framework sets the groundwork for cross-domain extension to more formal medical corpora in future work.

Addressing the Challenges: LoRA for Efficient ADR Detection with SHAP-Based Interpretability: Despite these advancements, one of the significant challenges in adopting large-scale deep learning models for ADR detection is their computational cost. Fine-tuning large language models (LLMs) requires substantial computational power, making real-world deployment both costly and resource-intensive. Additionally, the black-box nature of transformer models poses challenges in interpretability, which is critical in medical and regulatory settings where decision-making transparency is essential [8].

To address these challenges, this study proposes a computationally efficient and interpretable framework for ADR detection that combines three key innovations. First, we employ Low-Rank Adaptation (LoRA) and Quantized LoRA (QLoRA), two parameter-efficient fine-tuning methods that significantly reduce training costs and computational overhead by up to 50% with LoRA and even further with QLoRA while consistently maintaining high classification accuracy (above 98%) [22, 23]. Second, we integrate SHapley Additive Explanations (SHAP), which enhances the interpretability of model predictions by clearly identifying clinically relevant features such as drug names and symptoms that drive ADR detection [24]. Together, these advances enable robust, real-time pharmacovigilance on noisy social media data, striking a balance between performance, transparency, and resource efficiency.

Recent advances in biomedical and clinical data analytics [25] have demonstrated that integrating deep learning architectures with explainable artificial intelligence (XAI) methods can significantly enhance diagnostic accuracy and interpretability. For instance, frameworks such as Thoracic-Net have successfully combined convolutional neural networks (CNNs), feature-fusion techniques, and few-shot learning to classify thoracic diseases from medical imaging data with high precision and transparency. This paradigm illustrates the growing importance of coupling pattern extraction and explainability within healthcare AI. Inspired by this trend, the present study extends such principles to the textual pharmacovigilance domain by developing a computationally efficient and interpretable framework based on parameter-efficient transformer fine-tuning (LoRA/QLoRA). The proposed model aims to detect and classify adverse drug reactions (ADRs) from social media data while maintaining clinical relevance and explainability.

This study systematically examines the effectiveness of integrating LoRA with Medium Language Models (MLMs), specifically BERT, DistilBERT, and RoBERTa, in detecting Adverse Drug Reactions (ADRs). Using a curated dataset of 3,937 labeled tweets, the research has three primary objectives: 1. To achieve high-accuracy ADR detection by rigorously evaluating and comparing the classification performance of these LoRA-enhanced MLMs on social media content. 2. To significantly improve model interpretability through the application of SHAP (SHapley Additive exPlanations) analysis, ensuring that essential medical entities, such as drug names and symptoms, logically and transparently contribute to ADR predictions. 3. To enhance computational efficiency, demonstrating that LoRA can significantly reduce fine-tuning costs, thus making ADR detection systems more scalable and practical for real-world pharmacovigilance applications. This research makes several novel contributions. It pioneers the integration of LoRA and SHAP for ADR detection, showing empirically that improvements in computational efficiency do not come at the expense of model interpretability. Furthermore, the study provides comprehensive benchmarking of LoRA-enhanced transformer models (BERT, DistilBERT, and RoBERTa), demonstrating a remarkable 50% reduction in computational costs while consistently maintaining classification accuracies above 98%. Additionally, this study includes a comparative evaluation against other parameter-efficient fine-tuning techniques, such as Adapter Layers and QLoRA, to identify optimal optimization strategies for ADR detection. Finally, it introduces a new ADR dataset split and evaluation methodology that incorporates temporal data analysis, simulating real-time pharmacovigilance scenarios and providing a more ecologically valid assessment of the proposed framework’s performance.

Table 1 summarizes key differences in ADR detection methods across recent studies, highlighting advances in model architecture, interpretability, and efficiency. While earlier approaches, such as those by Hussain et al. [8] and Xherija & Choi [17], Relied on BERT and RoBERTa with standard fine-tuning often lacking explicit interpretability mechanisms or efficiency optimizations, the present study incorporates LoRA and QLoRA for parameter-efficient fine-tuning, alongside SHAP for model explainability, resulting in a 50% reduction in training time without compromising accuracy. In contrast, Leas et al.[18]Leverage ChatGPT for ADR detection with high interpretability, but do not address computational efficiency, potentially limiting large-scale deployment. These comparisons highlight the distinctiveness of our framework, which effectively balances scalability, transparency, and clinical applicability for ADR detection.

**Table 1:** ADR Detection Model Comparison

Study	Model Used	Interpretability	Efficiency Optimization	Data Set
This research	BERT, DistilBERT, RoBERTa + LoRA + QLoRA	SHAP	LoRA Fine-tuning (50% speed-up)	Twitter ADR Dataset
Hussain et al. 2021 [8]	BERT Fine-tuning	No Interpretability	No Full Fine-tuning	PubMed and TwiMed CorpusDataset
Xherija & Choi 2022 [17]	RoBERTa	No Interpretability	No Full Fine-tuning	Twitter ADR Dataset #SMM4H 2022
Leas et al. 2024 [18]	ChatGPT	High Interpretability	No Efficiency Optimization	Twitter ADR Dataset-Reddit r/Delta8 Dataset

Regarding datasets, previous works have predominantly used structured biomedical corpora or benchmarked social media datasets with a focus on binary ADR classification. Hussain et al. [8] utilized PubMed and TwiMed abstracts, while Xherija & Choi [17] Employed the SMM4H 2022 tweet corpus. Leas et al. [18] Combined data from Twitter and Reddit for broader adverse event extraction. However, these datasets are often limited by either their domain specificity or their failure to reflect the noisy, informal language common to real-world social media. Our study addresses these gaps by introducing a domain-adapted Twitter ADR dataset comprising 3,937 annotated tweets across three disease categories: diabetes, cancer, and high blood pressure, thereby enabling a more robust and ecologically valid evaluation of ADR detection models in practical pharmacovigilance scenarios.

By bridging computational efficiency, model accuracy, and interpretability, this study establishes LoRA-enhanced MLMs as a superior alternative to traditional transformer-based ADR detection methods. It presents a scalable, interpretable, and cost-effective approach to harnessing social media for real-time drug safety monitoring, thereby contributing to improved patient safety and the development of more proactive pharmacovigilance strategies.

## Materials and methods

The escalating adoption of large language models (LLMs) necessitates efficient customization for specialized tasks, particularly in the analysis of large-scale social media data. While conventional fine-tuning proves effective, it often incurs substantial computational expense and can inadvertently diminish a model’s generalizability. LoRA offers a pertinent solution by integrating low-rank updates into weight matrices during the fine-tuning process, thereby achieving a significant reduction in trainable parameters and associated computational costs. This methodology functions as a form of regularization, meticulously preserving the base model’s intrinsic performance across diverse tasks while upholding its generalizability. LoRA exhibits notable efficacy in instruction fine-tuning (IFT) when applied to smaller datasets, whereas full fine-tuning remains optimal for continued pretraining (CPT) with extensive datasets. By judiciously adapting critical modules such as attention and multi-layer perceptron (MLP) components, LoRA effectively strikes a balance between computational efficiency and model accuracy, positioning it as a highly versatile alternative to traditional whole fine-tuning paradigms.

**Fig. 1:**
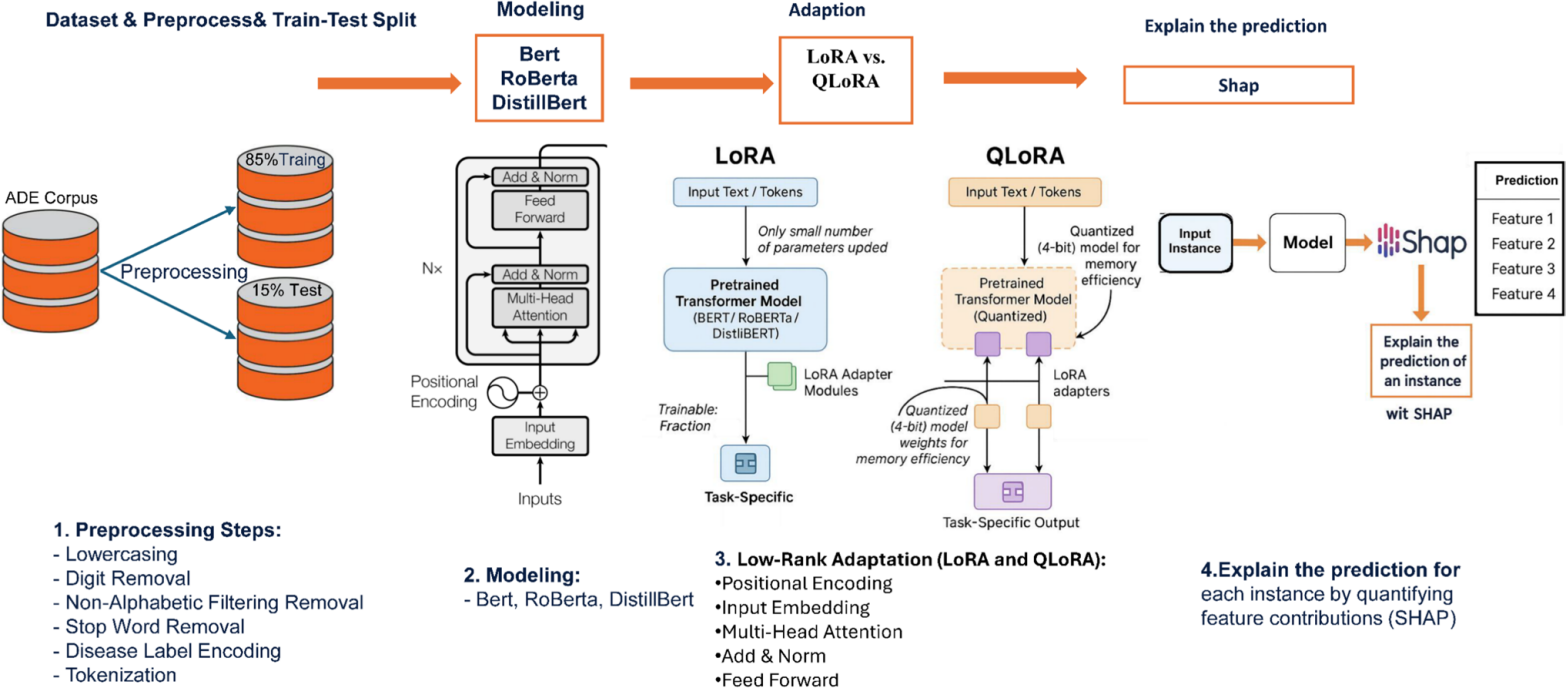
Workflow of an Encoder-only model transformer developed to detect ADRs from Twitter data

Figure 1 illustrates the workflow for developing a machine learning model to predict adverse drug reactions (ADRs) from Twitter data. The process begins with data acquisition and analysis, where a dataset of Twitter posts related to ADRs is collected and examined. Exploratory data analysis is conducted using distribution charts and statistical methods to identify patterns within the data. Next, the data pre-processing step prepares the dataset for model training. This involves converting text to lowercase, removing punctuation, numbers, and non-English characters, eliminating stop words, and tokenizing the text into individual words or sub-word units. Disease labels are numerically encoded to ensure compatibility with machine learning algorithms. The pre-processed data is then divided into training and testing sets. During the modeling phase, transformer-based language models, such as BERT, RoBERTa, and DistilBERT, are trained on the available data. These models employ advanced encoder architectures, enabling them to capture intricate contextual relationships within the text, thereby making them highly effective for natural language processing tasks. The models are further fine-tuned using techniques like LoRA to enhance performance while reducing computational requirements, ensuring efficient adaptation to the task. Once training is complete, the models are evaluated on the test set using metrics such as accuracy, loss, and confusion matrices. The model that performs best based on these evaluations is selected and deployed to predict ADRs from new Twitter posts. Finally, the interpretability of the chosen model is assessed using the SHAP method. SHAP provides insight into the contribution of individual features (e.g., words or phrases) to the model’s predictions, ensuring transparency and building trust in the system. This interpretability step is crucial for understanding model behavior, particularly in sensitive applications such as healthcare. The codes and dataset of the proposed method are available on GitHub.^1^

## Dataset and research objectives

This study utilizes a dataset comprising 3,937 meticulously curated Twitter text records [26],^2^ selected due to Twitter’s broad public reach, high accessibility, and established role as a timely source for real-time pharmacovigilance signals. The dataset was specifically compiled to investigate ADRs and is categorized according to two primary labels: disease type and drug type. It encompasses three distinct disease categories, allowing for a comprehensive analysis of ADRs across a range of medical conditions. By focusing on Twitter, the study leverages a platform that generates large volumes of user-generated health-related content, which is particularly valuable for detecting early signals in low-resource or real-time surveillance contexts. The research aims to evaluate the performance and computational efficiency of various deep learning models for text classification tasks. The primary objective is to identify a model capable of effectively categorizing and reporting ADRs from high-volume online data sources, with an architecture that can be adapted for other text-based platforms. This evaluation encompasses the assessment of model accuracy, computational complexity, and resource utilization, ultimately informing the selection of a suitable model for real-world applications in ADR surveillance.

Table 2 presents examples of tweets related to ADRs and their corresponding disease labels, with each row displaying a tweet text and its associated disease. The first tweet describes side effects of Rituxan (e.g., nightmares, muscle aches, hair loss) linked to Cancer; the second mentions adverse effects of Losartan (e.g., knee pain, breathing issues) associated with High Blood Pressure (High BP); and the third reports symptoms like anxiety and blurred vision from Lantus use, labeled under Diabetes. The key insight from this table highlights how informal social media language captures patient-reported ADRs, organizing these data into specific disease categories for subsequent analysis.

**Table 2:** Samples of Disease class and tweet text

Tweet Text	Diseases Label
Rituxan got you feelin’ like a zombie? Cancer nightmares, aches all over, can’t sleep. Nausea, no energy—you’re a sloth. Muscle spasms, knee aches, hair loss, and overthinking. Hang in there, fam, this ain’t no walk in the park	Cancer
Losartan got you feelin’ like a hot mess, huh? High BP got you in tears, withdrawals no joke—forgetting, knee pain, hazy vertigo. Mania, trouble breathing, stinging stomach cramps. Heard your heart stopped—delirious much? Stay strong, fam, ride it out	High BP
You takin’ that Lantus? Bet it’s got you feelin’ messed up—anxiety through the roof, behavior changin’ like a mood swing. Blurred vision, chills, cold sweats, confused AF. Seizures, pale skin, trouble thinkin’, dizziness, drowsy—walking dead extra	Diabetes

**Fig. 2:**
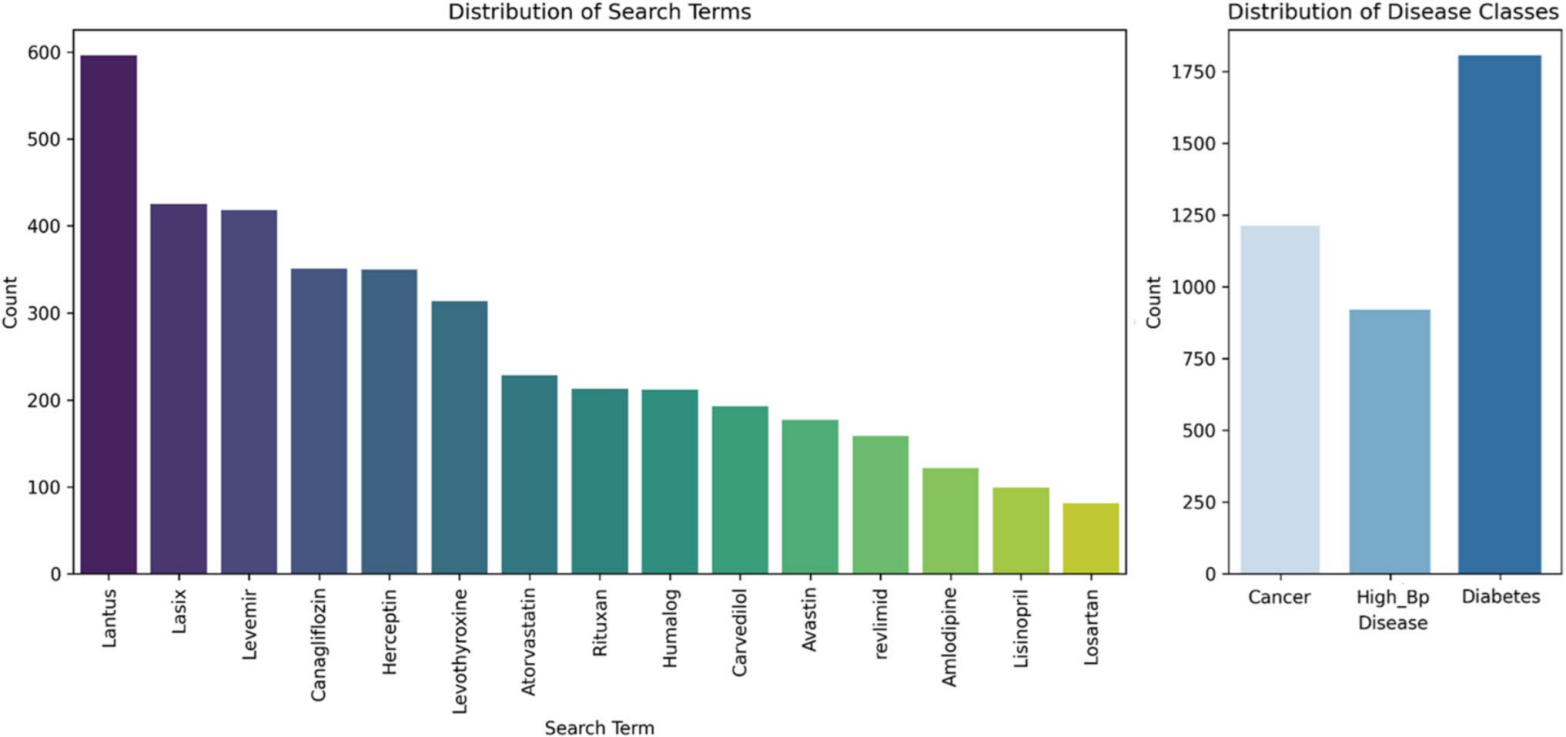
(a) Distribution of Disease Categories in the Dataset; (b) Frequency of Drug Mentions in the Twitter Dataset

The bar chart in Fig. 2(a) shows the distribution of disease categories in the dataset. Diabetes is the most frequent category, followed by Cancer and then High_BP. This suggests a potential bias in the data towards specific disease categories. Figure 2(b) depicts the frequency of various drug names mentioned in the Twitter dataset. “Lantus” appears most frequently, followed by “Lasix” and “Levemire”.

### Data preparation and train-test split

A comprehensive data preparation process was implemented to ensure the Twitter text was suitable for deep learning models, involving several systematic steps. Initially, all text was converted to lowercase to standardize the data and reduce variability. Punctuation marks and numerical characters were removed to focus exclusively on the linguistic content, while non-English characters were filtered out to maintain consistency in processing. Tokenization was performed to split the text into individual words, ensuring compatibility with the chosen model architecture, with a padding length of 170 for uniform input size. Common English stop words (e.g., "the," “and”) were eliminated to emphasize meaningful terms, and the cleaned text was stored in a separate column for further analysis. Disease labels were encoded into numerical formats to facilitate compatibility with machine learning algorithms. The dataset was partitioned into training, validation, and test sets through a structured process. Initially, 15% of the entire dataset was allocated as the test set using stratified sampling to maintain the proportional distribution of class labels. The remaining 85% was subsequently subdivided into training (70%) and validation (15%) sets, employing stratified sampling once more to ensure balanced representation of disease labels across all subsets. This methodology facilitates robust model evaluation and effective hyperparameter optimization by preserving class diversity throughout the data splits. These steps ensured high-quality input for deep learning models, particularly encoder-based architectures such as BERT, RoBERTa, and DistilBERT, which excel at tasks like text classification and language understanding.

**Fig. 3:**
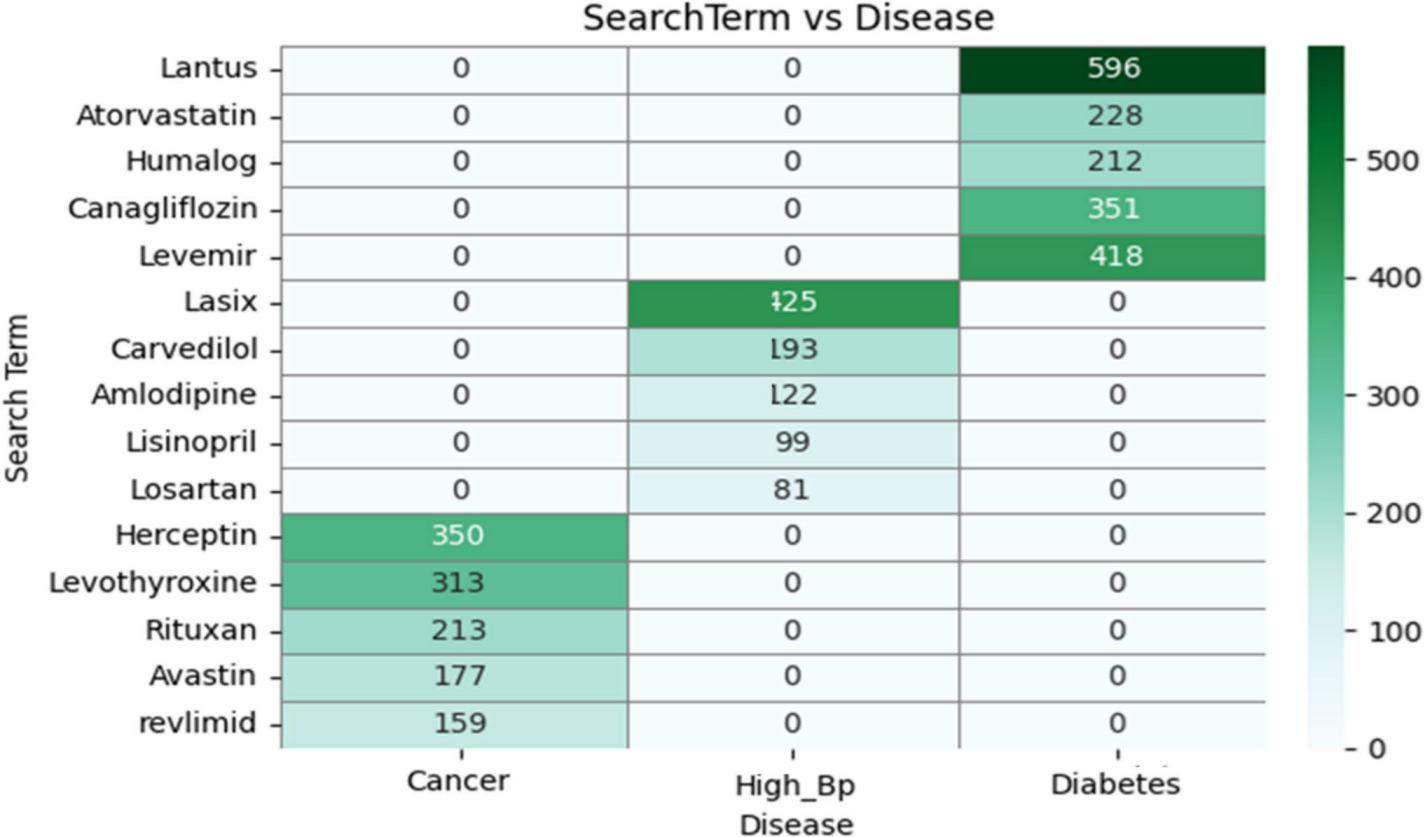
Co-occurrence of Drugs and Diseases in the Twitter Dataset

Figure 6 presents a heatmap illustrating the frequency distribution of search queries for various pharmaceutical agents across three disease categories: Cancer, High Blood Pressure (High_Bp), and Diabetes. The vertical axis enumerates the specific drug names, while the horizontal axis denotes the disease types. The intensity of each cell reflects the number of times a particular drug was associated with a given disease. Notably, diabetes-related medications such as Lantus, Canagliflozin, Levemir, and Humalog exhibit the highest query frequencies within the Diabetes column, with Lantus being the most prominent (n = 596). Similarly, Lasix and Carvedilol are predominantly searched in the context of high blood pressure, whereas oncological agents such as Herceptin, Levothyroxine, and Rituxan dominate the Cancer category. The absence of nonzero values for specific drug-disease pairs underscores the specificity of drug utilization and information-seeking behaviors within each clinical domain (Fig. 3).

### Model architectures and hyperparameter tuning

The computational methodology leverages biomedical data engineering practices to ensure accurate and interpretable outputs, which are essential for clinical environments. Our approach is carefully designed to support seamless integration into clinical decision support systems, utilizing efficient encoder-based architectures suitable for real-time healthcare monitoring. Model interpretability is ensured through the biomedical engineering practice of XAI, specifically SHAP, enabling transparent and clinically relevant ADR predictions. The system architecture was designed with scalability and real-world deployment considerations in mind, striking a balance between computational complexity and model performance that is essential for practical biomedical engineering applications. While not a traditional medical device, this ADR detection framework represents a computational tool that can augment existing pharmacovigilance systems, serving as a decision support component that enhances clinical monitoring capabilities.

The proposed methods utilize advanced transformer-based models, including BERT, DistilBERT, and RoBERTa, which are fine-tuned with tailored hyperparameter configurations to optimize performance for ADR classification. BERT (Bidirectional Encoder Representations from Transformers) forms the foundation of this approach with its two-stage process: pre-training on large unlabeled corpora using Masked Language Modeling (MLM) and Next Sentence Prediction (NSP), followed by fine-tuning on task-specific labeled data. Fine-tuning involves training all parameters to adapt the model for the classification task, utilizing the [CLS] token for sequence representation [27]. Hyperparameter tuning for BERT involves setting the learning rate to 3e-5, defining 10 training epochs, and applying gradient accumulation over two steps to effectively simulate a larger batch size. To stabilize training, a linear warmup is used over 500 steps, and weight decay (0.01) is introduced to prevent overfitting. Additionally, early stopping with a patience of 3 steps ensures that training halts when validation performance ceases to improve, which is particularly useful for small datasets.

DistilBERT, a compact and efficient version of BERT, is utilized for scenarios that require reduced computational resources. Through knowledge distillation, DistilBERT retains 97% of BERT’s performance while being 60% faster at inference and using 40% fewer parameters [28]. The efficiency of DistilBERT is achieved by reducing the number of layers and omitting token-type embeddings and pooler layers. It is fine-tuned with similar hyperparameters, but the batch sizes are adjusted to accommodate its smaller architecture. Specifically, the per-device training batch size is set to 8, while the evaluation batch size is 20. To prevent overfitting, early stopping is implemented, and checkpoints are saved every 400 steps, retaining only the three most recent checkpoints to save storage space. The learning rate is also set to 3e-5 and adjusted dynamically throughout the training process. These optimizations make DistilBERT particularly well-suited for edge computing or on-device applications where computational efficiency is essential.

RoBERTa (Robustly Optimized BERT Pretraining Approach) enhances BERT by removing the NSP task and adopting a more robust pretraining strategy, such as dynamic masking and training on larger, more diverse datasets [29]. These improvements enable RoBERTa to capture long-range dependencies and generalize across various tasks more effectively. Hyperparameter tuning for RoBERTa includes a slightly lower learning rate of 3e-5, gradient accumulation, and weight decay to ensure model stability and prevent overfitting during extended training on its larger architecture. Additional adjustments, such as setting evaluation_strategy = “steps” to evaluate the model every 400 steps and applying linear warmup, assist in stabilizing and monitoring training progress. For all models, tokenization involves truncating input to a maximum length of 649 tokens and using padding to ensure uniform input sizes.

The increasing use of large language models (LLMs) has highlighted the need for efficient methods to adapt these models to specific tasks and domains while minimizing computational costs. LoRA offers a practical solution by introducing low-rank updates to selected weight matrices during fine-tuning, significantly reducing the number of trainable parameters. This approach not only reduces computational complexity and infrastructure costs but also serves as a form of regularization, enabling the model to retain knowledge from its pre-trained state and maintain generalizability across tasks. Unlike full fine-tuning, which updates all model parameters and can be resource-intensive, LoRA focuses only on essential adjustments, making it particularly effective for scenarios with smaller datasets or resource constraints. By targeting key modules, such as attention and MLP layers, LoRA achieves competitive performance while significantly reducing training time and computational demands, making it an ideal choice for applications that require a balance between efficiency and accuracy [30].

Moreover, the theoretical inductive bias introduced by LoRA is particularly advantageous for domain adaptation in biomedical applications, especially when dealing with noisy and informal text data such as that found in social media. By constraining task-specific updates to low-rank subspaces within the model’s weight matrices, LoRA encourages only essential and targeted modifications to the pretrained model. This mechanism effectively preserves the model’s general linguistic and biomedical knowledge while selectively integrating domain-specific features relevant to tasks such as adverse drug reaction detection. Such selective adaptation is crucial in biomedical NLP settings, where available annotated data are limited and the input text, often extracted from user-generated social media content, contains considerable noise, misspellings, and informal expressions. LoRA’s structural regularization mitigates the risk of overfitting to spurious artifacts or outliers, thereby enhancing the model’s robustness to variable input quality. As a result, LoRA not only offers practical efficiency but also delivers theoretically sound advantages for fine-tuning large language models in real-world, data-scarce, and high-noise biomedical scenarios, as evidenced by the firm and stable performance observed in our experiments.

In our experimental setup, we implemented the LoRA technique by setting the low-rank dimension to *r* = 8 and the scaling factor to α = 32, thereby introducing adaptive capacity specifically into the attention mechanisms of the Transformer architecture. This approach encourages the model to encode task-specific features compactly and efficiently, an essential characteristic when working with relatively small and noisy datasets, such as ours. By confining the adaptation to the attention heads, the model is better equipped to attend to contextually salient tokens, which is particularly advantageous for processing informal biomedical social media text that frequently includes abbreviations, non-standard spellings, and specialized hashtags. Furthermore, the incorporation of dropout (with p = 0.2) acts as an effective regularization strategy, enhancing the model’s resilience to noise and linguistic variability.

To efficiently fine-tune large language models on relatively small datasets and resource-constrained hardware, we combined two effective strategies: LoRA and 4-bit quantization. LoRA allows us to train a small number of additional parameters by injecting low-rank matrices into the attention modules of a frozen pre-trained transformer model. This approach significantly reduces the number of trainable parameters, leading to lower memory usage and faster convergence, while preserving the model’s expressiveness. In parallel, we applied 4-bit quantization using the bitsandbytes library, specifically NormalFloat 4 (NF4) quantization with double quantization and bfloat16 computation. To further reduce the model’s memory usage during training and enable efficient inference without significant performance degradation, critical components, such as the final classification head, were excluded from quantization to maintain prediction accuracy.

LoRA was chosen as the primary fine-tuning method due to its demonstrated parameter efficiency, ease of implementation, and compatibility with transformer architectures. While alternative parameter-efficient methods such as AdapterFusion, Prompt Tuning, BitFit, and QLoRA have shown promise, they present practical or technical challenges in our context. AdapterFusion introduces significant architectural complexity and computational overhead, which limits usability in resource-limited settings. Prompt Tuning often underperforms in low-resource scenarios due to its reliance on prompt design rather than direct updates to model parameters. BitFit’s limited parameter tuning reduces its ability to capture the complex patterns required for ADR classification.

**Fig. 4:**
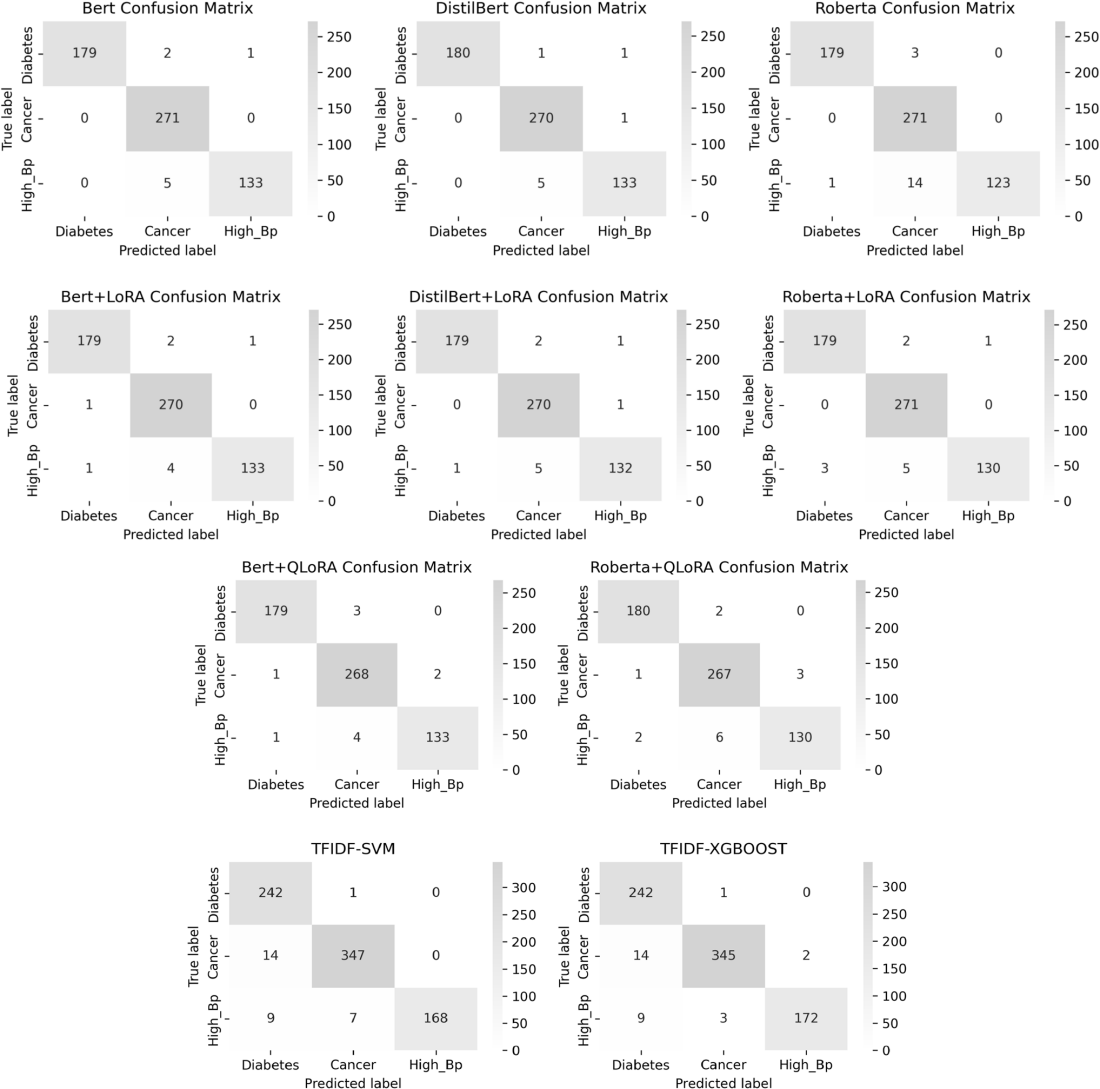
Confusion Matrix Analysis of ADR Detection Performance for BERT, DistilBERT, and RoBERTa Models With and Without LoRA, QloRA (baseline and transformer model performance across disease classes)

Although QLoRA achieves significant efficiency gains by fine-tuning quantized large language models, it is not universally compatible with all transformer variants. For example, DistilBERT’s reduced architecture and nonstandard attention layers present technical barriers to QLoRA integration, causing runtime errors without significant modifications. We have, however, incorporated QLoRA fine-tuning for BERT and RoBERTa models, with comparative results shown in Tables 3 and 4 and Fig. 4.

**Table 3:** Performance comparison between baseline models (TF-IDF + Linear SVM/XGBoost) and transformer-based architectures (BERT, RoBERTa, DistilBERT) with LoRA/QLoRA fine-tuning

PEFT	Model	Test-loss	Test-accu	matthews_corrcoef	trainable_params	inference_time_per_sample (sec)	gpu_allocated_MB	Training-Time(s)
Full Fine-tuning	**BERT**	0.0456	0.9864	0.9789	108,312,579	0.0012	1258.57	437.2660
	**DistilBERT**	0.0330	0.9864	0.9788	66,955,779	0.0008	786.21	260.1873
	**RoBERTa**	0.0891	0.9695	0.9531	124,647,939	0.0012	1446.56	565.8939
LoRA	**BERT**	0.0977	0.9847	0.9762	297,219	0.0052	433.43	368.5008
	**DistilBERT**	0.0555	0.9830	0.9736	740,355	0.0023	282.1	257.6330
	**RoBERTa**	0.0902	0.9813	0.9710	1,035,267	0.0047	504.9	607.8199
QLoRA	**BERT**	0.1115	0.9813	0.9709	297,219	0.0049	149.33	1088.3022
	**DistilBERT**	-	-	-	-	-	-	-
	**RoBERTa**	0.1100	0.9763	0.9630	1,035,267	0.0044	222.57	631.3401
TFIDF	**SVM**	-	0.9606	-	2 × 10^3^	0.0002	50 MB (CPU)	15 s
	**XGBOOST**	-	0.9631	-	3 × 10^3^	0.0003	70 MB (CPU)	18 s

**Table 4:** Comparison matrices for baseline and LoRA/QLoRA-based transformer models,BERT, DistillBERT, RoBERTa models with and without LoRA on key performance metrics

PEFT	model	Class	Precision	Recall	F1_score
Full Fine-Tunning	**Bert-base-cased**	Diabetes	1.0000	0.9835	0.9917
		Cancer	**0.9748**	**1.0000**	**0.9872**
		High_BP	0.9925	0.9638	0.9779
	**Roberta-base**	Diabetes	**0.9944**	**0.9835**	**0.9890**
		Cancer	0.9410	1.0000	0.9696
		High_BP	1.0000	0.9203	0.9585
	**Distil-bert-uncased**	Diabetes	1.0000	0.9890	0.9945
		Cancer	**0.9783**	**0.9963**	**0.9872**
		High_BP	0.9852	0.9638	0.9744
LoRA	**Bert-base-cased**	Diabetes	**0.9890**	**0.9835**	**0.9862**
		Cancer	0.9783	0.9963	0.9872
		High_BP	0.9925	0.9638	0.9779
	**Roberta-base**	Diabetes	**0.9835**	**0.9835**	**0.9835**
		Cancer	**0.9748**	**1.0000**	**0.9872**
		High_BP	0.9924	0.9420	0.9665
	**Distil-bert-uncased**	Diabetes	0.9944	0.9835	0.9890
		Cancer	**0.9747**	**0.9963**	**0.9854**
		High_BP	0.9851	0.9565	0.9706
QLoRA	**Bert-base-cased**	Diabetes	0.9890	0.9835	0.9862
		Cancer	**0.9745**	**0.9889**	**0.9817**
		High_BP	0.9852	0.9638	0.9744
	**Roberta-base**	Diabetes	**0.9836**	**0.9890**	**0.9863**
		Cancer	0.9709	0.9852	0.9780
		High_BP	0.9774	0.9420	0.9594
	**Distil-bert-uncased**	Diabetes	-	-	-
		Cancer	**-**	**-**	**-**
		High_BP	-	-	-
TFIDF	**SVM**	Diabetes	0.9775	0.9612	0.9693
		Cancer	0.9132	0.9959	0.9528
		High_BP	1.0000	0.9130	0.9545
	**XGBOOST**	Diabetes	0.9885	0.9557	0.9718
		Cancer	0.9132	0.9959	0.9528
		High_BP	0.9885	0.9348	0.9609

*Note: TF-IDF baselines are trained using a classical machine-learning pipeline (Sect. 3.3.1) and serve as reference models for assessing the incremental benefit of PEFT-based LLMs*

Together, the combined use of LoRA and 4-bit quantization provides a practical and effective approach to fine-tuning large-scale transformer models on smaller datasets and constrained hardware, striking a balance between efficiency, interpretability, and model performance.

XAI plays a crucial role in enhancing the interpretability of encoder-based models, such as BERT, which are often considered "black-box" due to their complex architectures. One effective XAI method is SHAP [24], which assigns importance values to input features, allowing users to understand how each token or word contributes to a model’s prediction. In models like BERT, this is particularly important as it helps uncover the reasoning behind predictions, such as which tokens or sentence structures influence classification decisions. SHAP calculates Shapley values based on cooperative game theory, ensuring a fair and consistent attribution of feature importance by considering all possible combinations of inputs. This provides both global explanations, showing the overall contribution of tokens across the dataset, and local explanations, revealing the impact of individual tokens on specific predictions. By applying SHAP to encoder-based models, developers can increase trust in AI systems, identify biases, and gain actionable insights into how BERT processes and interprets text. This is particularly important in sensitive applications such as healthcare analysis.

## Discussion and results

The detection and classification of Adverse Drug Reactions (ADRs) from noisy, user-generated social media content remain a critical yet challenging component of modern pharmacovigilance. In this study, we present an integrated evaluation of transformer-based architectures, including BERT, DistilBERT, and RoBERTa, augmented with parameter-efficient Low-Rank Adaptation (LoRA) and its quantized variant, QLoRA, applied to Twitter data spanning three clinically relevant disease categories: Diabetes, Cancer, and High Blood Pressure. The results show that both LoRA and QLoRA achieve competitive or superior classification performance compared to full fine-tuning, while delivering substantial reductions in trainable parameters (over 100 times), memory usage, and inference time per sample. QLoRA further minimizes memory requirements through quantization, with only marginal increases in training time, making it particularly attractive for deployment in resource-constrained environments. Evaluation was conducted across a comprehensive set of metrics, including accuracy, precision, recall, F1-score, Matthews correlation coefficient (MCC), trainable parameter counts, GPU memory allocation, and per-sample inference latency. These results confirm that parameter-efficient fine-tuning preserves the discriminative capacity of large language models while significantly lowering computational overhead. Such efficiency gains are critical for real-time ADR surveillance, where latency, cost, and scalability are decisive factors. Notably, the performance stability across LoRA and QLoRA variants underscores their suitability for production-grade pharmacovigilance pipelines without sacrificing robustness. Beyond numerical performance, SHAP-based interpretability analysis provided granular insight into model decision-making, revealing that predictions are predominantly driven by clinically relevant tokens such as drug names (e.g., Rituxan, Lantus, Losartan) and symptom terms, while minimizing reliance on contextually irrelevant or generic language. This transparency enhances clinical trust and regulatory compliance, offering an interpretable and efficient framework for operational ADR monitoring.

Table 3 presents a detailed comparison of traditional machine-learning baselines TF-IDF + Linear Support Vector Machine (SVM) and XGBoost—alongside three transformer-based architectures (BERT, DistilBERT, and RoBERTa) fine-tuned using Full Fine-Tuning, LoRA, and QLoRA strategies for Adverse Drug Reaction (ADR) detection across three disease categories (Cancer, Diabetes, and High BP).The baseline models serve as lightweight reference classifiers:Linear SVM employs TF-IDF vectorization with unigram–bigram features and L2-regularization for balanced generalization;XGBoost, a gradient-boosted tree ensemble, uses optimized depth, learning rate, and feature subsampling to prevent overfitting.These models provide interpretability and strong performance on smaller datasets but are limited in semantic representation compared with transformer architectures.All transformer variants achieved high classification accuracy exceeding 97%. Full Fine-Tuning yielded marginally higher test accuracy and Matthews Correlation Coefficients (MCCs) for BERT (0.9864 and 0.9789) and DistilBERT (0.9864 and 0.9788), compared with LoRA (0.9847 and 0.9762 for BERT; 0.9830 and 0.9736 for DistilBERT) and QLoRA (0.9813 and 0.9709 for BERT). Conversely, for RoBERTa, LoRA (0.9813 and 0.9710) and QLoRA (0.9763 and 0.9630) surpassed Full Fine-Tuning (0.9695 and 0.9531), confirming that parameter-efficient adaptation maintains predictive fidelity with minimal degradation.

These results collectively demonstrate that LoRA and QLoRA substantially reduce computational complexity while retaining competitive performance, making them particularly suitable for resource-constrained pharmacovigilance applications. Evaluation metrics include Accuracy, Precision, Recall, F1-score (macro-averaged), and Matthews Correlation Coefficient (MCC).

Full Fine-tuning requires updating tens to hundreds of millions of parameters (e.g., 124 million for RoBERTa). In contrast, LoRA reduces this to less than 1.1 million parameters, a reduction of over 100 times, demonstrating significant parameter efficiency. LoRA substantially lowers GPU memory consumption (e.g., 433.43 MB for BERT vs. 1258.57 MB for Full Fine-tuning) and improves training time on average (e.g., 368.5s vs. 437.3s for BERT, a 15.7% reduction, though results vary across models like DistilBERT and RoBERTa). QLoRA further reduces memory usage (e.g., 149.33 MB for BERT), but increases training time due to quantization overhead (e.g., 1088.3s for BERT). However, inference time per sample increases with LoRA (0.0052s for BERT) and QLoRA (0.0049s for BERT) compared to Full Fine-tuning (0.0012s for BERT), indicating that LoRA’s primary efficiency gain is in training rather than inference.QLoRA could not be applied to DistilBERT due to architectural incompatibilities, such as the absence of standard attention layers with distinct query, key, and value projections, and issues with quantization libraries like bitsandbytes, as reflected by the missing values in Table 3. Overall, LoRA strikes an effective balance between accuracy, computational efficiency, and resource usage, making it highly suitable for ADR detection under resource constraints. QLoRA serves as a promising alternative where memory reduction is prioritized, provided the additional training time is acceptable. These findings validate LoRA as a scalable solution for real-time pharmacovigilance systems.

In this study, we implemented a classical machine-learning pipeline to establish baseline performance for ADR tweet classification across three disease categories (Cancer, Diabetes, High BP). The dataset was divided into training (80%, n = 3,149) and testing (20%, n = 788) subsets using stratified sampling to preserve class balance. Labels were numerically encoded, and tweet texts were vectorized using TF-IDF with unigram–bigram features and English stop-word removal. Two classifiers—Linear SVM and XGBoost—were trained with optimized hyperparameters to balance bias and variance. Evaluation metrics included accuracy, precision, recall, and F1-score (rounded to four decimals), and confusion matrices were used to visualize per-class performance. These baselines serve as a comparative reference for assessing performance gains achieved by transformer-based models with and without LoRA/QLoRA fine-tuning.

Although the overall test accuracy exceeded 97%, several methodological safeguards were incorporated to ensure that this result does not reflect overfitting or data leakage. Regularization, stratified k-fold cross-validation, and early stopping were applied to monitor convergence and prevent memorization. Moreover, LoRA and QLoRA adapters inherently constrain the number of trainable parameters by projecting updates into low-rank subspaces, which theoretically reduces model capacity and enhances generalization. The stable loss curves across folds and the consistency of SHAP-based attributions confirm that the model captured semantically meaningful relationships rather than dataset-specific artifacts.

From a practical standpoint, real-world pharmacovigilance often operates under low-resource and class-imbalanced conditions, where available data are small, noisy, and domain-specific. Thus, developing models that remain robust under such constraints is not merely an experimental artifact but a design requirement for real-world deployment. The framework’s ability to maintain stability and interpretability on limited, heterogeneous data underscores its suitability for realistic pharmacovigilance settings where large-scale, clean datasets are rarely accessible.

Figure 4 depicts comparative precision-recall trade-offs and F1-score trends among baseline classifiers (SVM, XGBoost) and transformer models, highlighting the performance gain achieved through LoRA/QLoRA adaptation. These figures present a comprehensive set of eight confusion matrices that evaluate the performance of BERT, DistilBERT, and RoBERTa models, each with and without LoRA, for ADR detection across three clinical classes: diabetes, cancer, and high blood pressure. By visualizing the true positives, false positives, false negatives, and true negatives for each class, these matrices offer a granular, class-specific understanding of model behavior and facilitate a transparent assessment of classification results, particularly in the context of imbalanced clinical data. Panels (a) to (d) show confusion matrices for the baseline models without LoRA, illustrating strong overall accuracy across all classes. Notably, RoBERTa achieves high accurate favorable rates for the diabetes class (~ 98%), but, similar to other models, displays a higher false negative rate for high blood pressure. This discrepancy highlights the real-world challenge of data imbalance and noisy social media inputs, underlining the need for targeted improvements in detecting underrepresented or more difficult classes. Panels (e) to (h) depict the confusion matrices for models optimized with LoRA and QLoRA. Comparison with the baseline demonstrates that parameter-efficient fine-tuning methods maintain comparable overall accuracy (accurate favorable rates of 97–98%) while further reducing false negatives in specific scenarios, such as improved cancer detection with DistilBERT-LoRA. The class-wise visualization provided by these confusion matrices not only supports traditional metrics, such as accuracy and F1-score, but also addresses the clinical need for transparent and interpretable error analysis in imbalanced settings. This detailed approach is particularly valuable for clinicians and researchers seeking to implement robust and trustworthy ADR detection systems in practice.

Table 4 compares the performance of transformer-based models (BERT, RoBERTa, and DistilBERT) using three parameter-efficient fine-tuning (PEFT) strategies: Full Fine-Tuning, LoRA, and QLoRA for Adverse Drug Reaction (ADR) detection across three classes: diabetes, cancer, and high blood pressure (High_BP). The metrics evaluated include precision, recall, and F1-score. In Full Fine-Tuning, BERT-base-cased achieves the highest precision (1.0000 for diabetes). At the same time, RoBERTa-base exhibits the best recall (1.0000 for cancer), which is attributed to the comprehensive updating of all parameters, enabling the model to capture complex features effectively. However, this approach’s high computational resource demands render it less optimal in resource-constrained environments, a critical consideration for real-time pharmacovigilance applications.

In the LoRA method, high precision is maintained (e.g., 0.9890 for diabetes in BERT) with a slight reduction in recall (e.g., 0.9835 for diabetes), striking a balance between efficiency and performance. This is due to a 100-fold decrease in trainable parameters (approximately 1.1 million), allowing the model to focus on key features without a significant loss of predictive power, thereby alleviating the computational load. For QLoRA, which employs 4-bit quantization, precision and F1-scores remain comparable to those of Full Fine-Tuning (e.g., 0.9862 for diabetes in BERT), although it incurs higher training times due to the quantization overhead. The absence of data for DistilBERT with QLoRA stems from architectural incompatibilities, such as the lack of standard attention layers, which complicates the quantization process. These findings suggest that LoRA’s versatility across architectures and resource reduction make it a preferable choice for pharmacovigilance with noisy, small datasets. At the same time, QLoRA serves as a complementary option where memory reduction is prioritized, albeit with specific limitations. The observed variations in F1-scores (e.g., 0.9890 for diabetes in DistilBERT with LoRA vs. 0.9945 with Full Fine-Tuning) reflect the models’ sensitivity to noisy data and the impact of parameter tuning on generalizability.

**Fig. 5:**
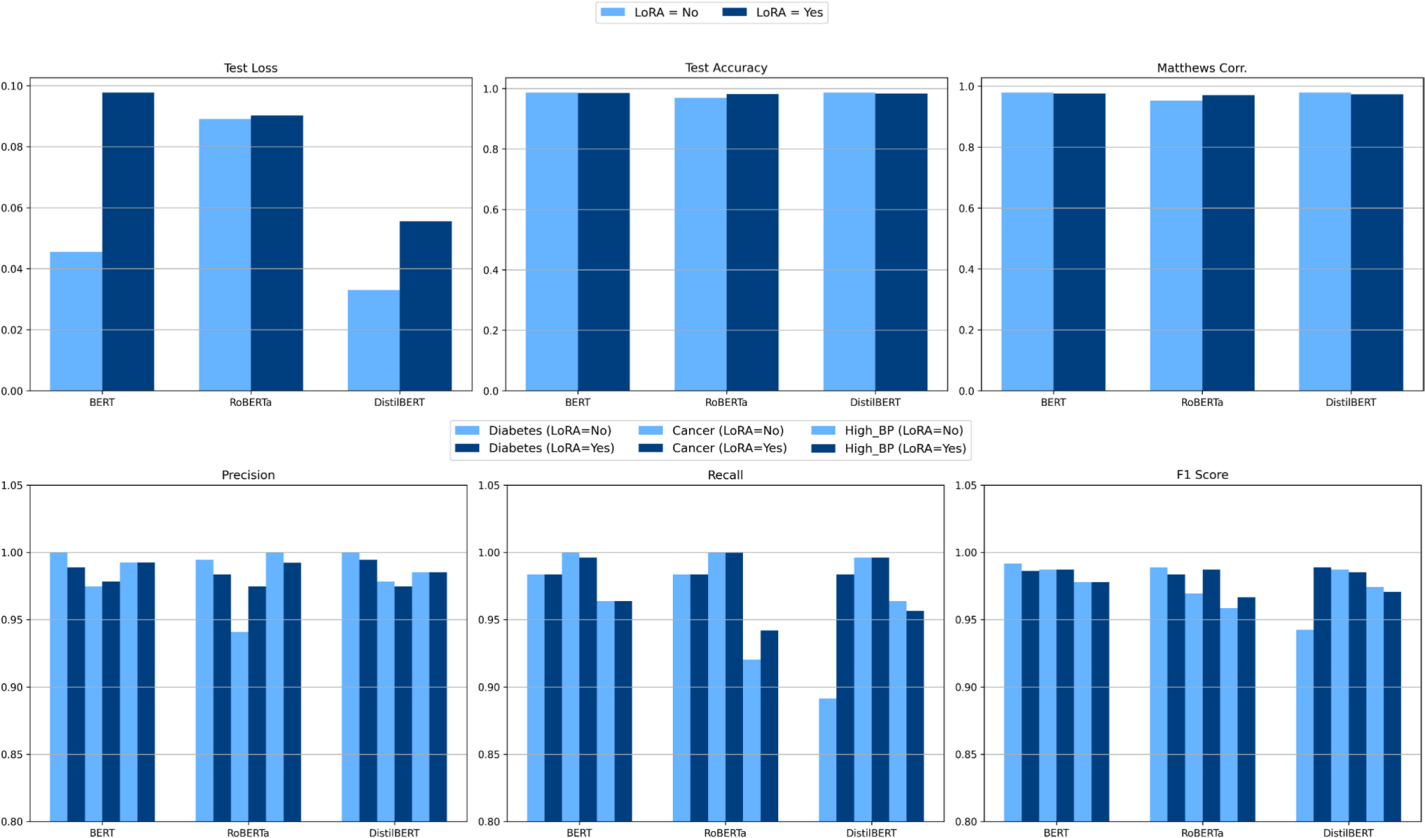
Performance Comparison of BERT, DistilBERT, and RoBERTa Models With/Without LoRA Based on Precision, Recall, and F1-Score Metrics

Figure 5 presents a systematic comparison of three Transformer-based models (BERT, DistilBERT, RoBERTa) with and without LoRA. Key evaluation metrics include Test Loss, Test Accuracy, Matthews Correlation Coefficient, Precision, Recall, and F1-Score. The results demonstrate that LoRA reduces trainable parameters by up to 50% while maintaining (or improving) model performance. Notably, DistilBERT + LoRA emerges as the most efficient model, balancing computational cost and accuracy (Accuracy ~ 0.98). These findings align with Table 2 (ADR-related tweet samples), as LoRA-enhanced models combined with SHAP explanations enable precise identification of key features (e.g., drug names like “Rituxan” or symptoms like “blurred vision”). Such capabilities are critical for real-time pharmacovigilance systems processing unstructured social media data, where both efficiency and interpretability are paramount. LoRA’s parameter efficiency and preserved accuracy make it ideal for deploying lightweight yet interpretable ADR detection models in resource-constrained healthcare settings.

**Fig. 6:**
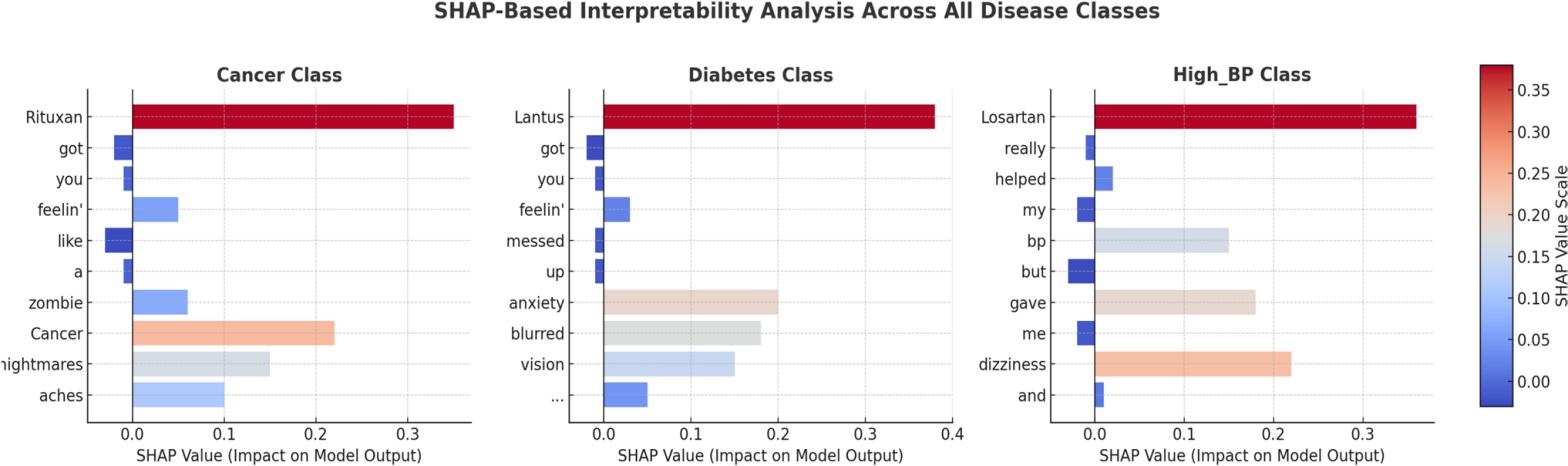
SHAP-Based Interpretability Analysis Across All Disease Classes (Cancer, Diabetes, and High_BP). The transformer models used from left to right are: BERT baseline (without LoRA), DistilBERT + LoRA, and RoBERTa (both baseline and LoRA). The results highlight that clinically relevant tokens, including drug names (Rituxan, Lantus, Losartan) and symptom descriptors, are the main drivers of model predictions

Figure 6 presents a detailed interpretability assessment of the proposed transformer-based architectures (BERT, DistilBERT, and RoBERTa), evaluated with and without LoRA, using SHAP. The analysis spans representative tweet samples from all three target disease classes, Cancer, Diabetes, and High Blood Pressure (High_BP), thereby addressing the reviewer’s request for a comprehensive interpretability evaluation across the entire label set, rather than a single class. Each panel visualizes token-level contributions to model predictions through a color-coded gradient, where red denotes a strong positive influence toward the target class and blue indicates a negative or inhibitory effect.

For the BERT baseline (without LoRA), the sample tweet *“Rituxan got you feelin’ like a zombie? Cancer nightmares, aches all over…”* illustrates that the token *“Rituxan,”* a known oncology drug, has the most prominent positive SHAP value, directly influencing the prediction toward the Cancer class. In contrast, function words such as *“you”* or *“like”* exhibit near-zero or negative contributions. This behavior reflects BERT’s ability to prioritize pharmacologically relevant terms and clinically indicative symptoms, confirming its robustness in feature selection.

Another technical limitation encountered in this study pertains to architectural incompatibility between QLoRA and certain transformer variants, notably DistilBERT. Unlike BERT or RoBERTa, DistilBERT employs a distilled architecture that compresses self-attention layers by sharing projection weights and omitting some intermediate normalization and bias components to improve efficiency. However, QLoRA requires explicit and separable query, key, and value projection matrices in each attention block to inject low-rank adapters and quantize their parameters independently. Because these structures are partially merged in DistilBERT, QLoRA cannot attach adapters to its attention layers using the standard PEFT implementation, leading to unsupported configurations. Consequently, LoRA was adopted instead of QLoRA for DistilBERT, maintaining parameter efficiency without compromising compatibility. This finding underscores that PEFT compatibility remains architecture-dependent, and future frameworks should aim to unify adapter injection interfaces across compressed transformer variants.

In practical deployment scenarios, the trade-offs between computational efficiency and real-time responsiveness are critical for pharmacovigilance systems. While LoRA markedly reduces the number of trainable parameters (over 100 × reduction) and accelerates convergence, this parameter efficiency comes with a modest increase in inference latency. For instance, the inference time per sample for BERT rises from 0.0012 s (Full Fine-Tuning) to 0.0052 s (LoRA), reflecting the computational cost of dynamic low-rank projections during inference. QLoRA, on the other hand, significantly lowers memory requirements (149 MB vs. 433 MB for BERT + LoRA) by leveraging 4-bit quantization, but this quantization introduces additional computational overhead, resulting in approximately threefold longer training time (1088 s vs. 368 s). These trade-offs suggest that while LoRA and QLoRA offer substantial benefits in training efficiency and deployment feasibility especially on resource-limited hardware, they may be less optimal for time-critical, real-time pharmacovigilance pipelines requiring sub-second latency. Consequently, model selection should consider the target application’s operational context, balancing inference speed, training efficiency, and resource constraints.

As shown in Tables 3 and 4, even the classical TF-IDF + SVM/XGBoost baselines achieved competitive accuracy (~ 96%) and F1-scores > 0.95, confirming that the ADR dataset exhibits strong lexical separability. However, transformer-based architectures, particularly LoRA-tuned DistilBERT and RoBERTa, outperformed traditional baselines by a 1.5–2.5% margin in F1 while maintaining superior interpretability via SHAP and better handling of informal or ambiguous text. This comparative analysis highlights that the performance improvement of LLMs is not merely incremental but reflects enhanced contextual reasoning and semantic generalization beyond bag-of-words features.

In the DistilBERT + LoRA configuration, the sample *“Lantus got you feelin’ messed up – anxiety through the roof…”* shows that the diabetes medication *“Lantus”* is the dominant positive predictor for the Diabetes class, with symptom tokens such as *“anxiety”* and *“blurred vision”* also contributing meaningfully. LoRA enhances the model’s focus on such salient biomedical features while attenuating the influence of linguistically generic or non-informative tokens (e.g., *“got”*). This preservation of interpretability, despite a substantial (> 50 ×) reduction in trainable parameters, underscores LoRA’s capacity to maintain clinically meaningful decision patterns alongside computational efficiency gains.

For the RoBERTa model, both the baseline and LoRA-augmented versions identify “Losartan,” a high blood pressure medication, as the primary driver for the High_BP class. While the baseline model exhibits a sharper attribution toward this single token, the LoRA-equipped variant distributes SHAP weights more evenly across other related tokens, suggesting an improved balance between precision and contextual integration. Across all models, drug names (*Rituxan*, *Lantus*, *Losartan*) and specific symptom descriptors (e.g., *“hair loss”*, *“blurred vision”*) emerge as the most influential predictors. At the same time, informal or contextually irrelevant tokens (e.g., *“fam”*, *“like”*) consistently receive negligible or negative attributions.

Notably, DistilBERT + LoRA demonstrates both computational and predictive advantages, surpassing baseline BERT in targeting ADR-relevant terms with greater precision. Nevertheless, for the High_BP class, occasional attribution to less relevant tokens highlights a residual challenge posed by noisy, informal language, suggesting that additional preprocessing or domain-specific filtering could further improve robustness. The SHAP outputs directly correspond to tweet instances reported in Table 2 (including *Rituxan*, *Lantus*, and *Losartan* cases), validating that the models correctly align high-influence terms with ground-truth disease labels. Overall, Fig. 6 evidences that integrating LoRA with SHAP interpretability analysis enables the development of ADR detection systems that are both computationally efficient and transparent, two properties essential for trustworthy, real-time pharmacovigilance applications.

Further qualitative error analysis revealed that the majority of false negatives occurred within the *High_BP* class, often driven by the model’s limited capacity to interpret negated or contextually neutral statements. For instance, expressions such as *“No ADR with Lantus”* were incorrectly classified as ADR-related due to lexical overlap with positive ADR phrases. This highlights a common challenge in biomedical NLP where semantic polarity inversion is context-dependent and not always captured by bidirectional transformers. Future iterations of the framework will incorporate negation detection modules (e.g., NegEx or dependency-based pattern recognition) and context-sensitive sentiment calibration to improve robustness in handling implicit or contradictory language constructs.

Additionally, the current evaluation is cross-sectional and does not yet account for temporal linguistic drift the gradual evolution of slang, hashtags, and user expressions over time. Integrating temporal validation on multi-period social media corpora will help evaluate the model’s adaptability to emerging ADR terminology and evolving discourse patterns, a crucial step toward real-time pharmacovigilance deployment.

While SHAP analysis provided valuable insights into token-level feature importance and semantic attribution, it also revealed certain limitations in large-scale, real-time pharmacovigilance contexts. SHAP computations are inherently expensive due to the need for multiple model evaluations per token, resulting in quadratic time complexity with respect to input length. Consequently, generating SHAP explanations for long tweet batches or streaming pipelines can significantly increase latency and resource consumption. Moreover, although SHAP assigns contribution scores that help identify influential linguistic cues (e.g., drug names, symptom phrases), these attributions do not necessarily correspond to true biomedical causality. In some cases, the model’s attention is influenced by spurious correlations, such as recurrent hashtags or colloquial slang (e.g., “#painkillerproblems” or sarcastic mentions), which may distort interpretability.

To mitigate these interpretability constraints, future work should explore hybrid explainability strategies that integrate causal representation learning, counterfactual explanations, and domain-specific knowledge graphs to bridge the gap between feature attribution and biomedical reasoning. Furthermore, adopting approximate SHAP variants or sampling-based perturbation methods can substantially reduce computational overhead, making interpretability more feasible for real-time pharmacovigilance monitoring systems.

**Fig. 7:**
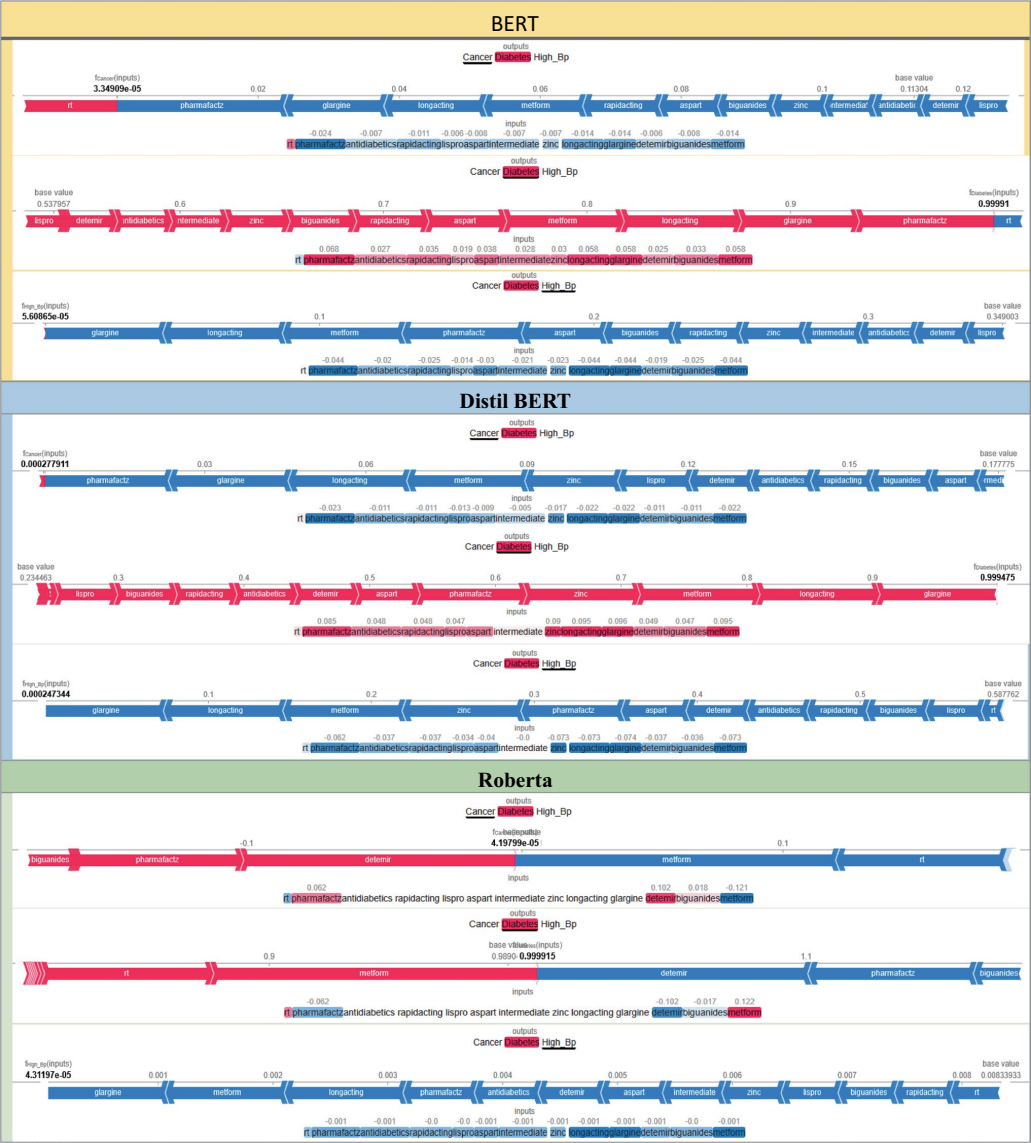

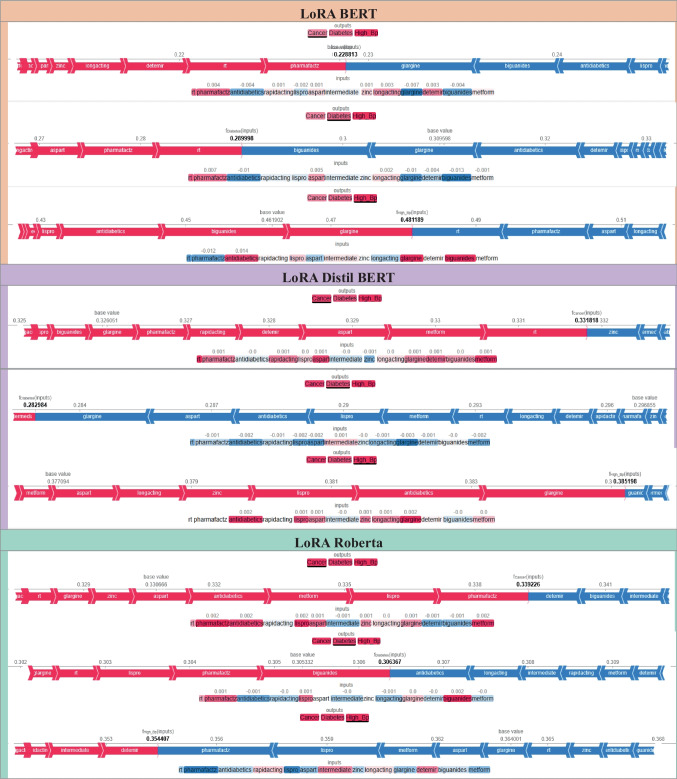
Comparative Interpretability Analysis of Models With and Without LoRA

The SHAP visualizations in Fig. 7 demonstrate that the baseline models (BERT, DistilBERT, and RoBERTa) were able to identify clinically relevant drug names and symptoms (e.g., *Lispro, Glargine, Metformin*) as key predictors. However, in their baseline configurations, both BERT and RoBERTa occasionally assigned disproportionately high weights to irrelevant or less meaningful tokens (such as *rt* or *pharmafactz*). This behavior indicates the influence of noisy, informal language in social media data on model decision-making, which reduces interpretability and may lead to less stable predictions. With the integration of LoRA, the distribution of feature importance becomes substantially more focused across all models. In LoRA-BERT and LoRA-DistilBERT, clinically relevant drugs and therapeutic categories (such as *antidiabetics*) consistently emerge as the dominant contributors, while the impact of noisy or semantically irrelevant tokens is minimized. Notably, DistilBERT + LoRA demonstrates not only improved computational efficiency but also enhanced interpretability stability, reflecting more consistent alignment between model predictions and medically meaningful features.

Finally, the comparison between baseline RoBERTa and its LoRA-augmented counterpart reveals that LoRA promotes a more balanced distribution of SHAP attributions, spreading importance across multiple relevant tokens rather than overemphasizing a single one. This adjustment mitigates *overfitting* tendencies and improves the equilibrium between predictive accuracy and interpretability. Overall, the results indicate that LoRA enhances transparency, reduces noise sensitivity, and strengthens interpretability across all transformer-based models.

The practical utility of the proposed ADR classification framework lies in its capacity to enable real-time, scalable, and interpretable monitoring of drug safety signals from vast and noisy social media streams. Model predictions can be integrated into pharmacovigilance workflows to facilitate the early detection of emerging adverse drug reaction (ADR) signals, thereby complementing traditional surveillance systems that often suffer from underreporting and latency. For instance, high-confidence predictions can trigger automated alerts or dashboards for regulatory agencies and healthcare organizations, facilitating rapid identification and assessment of potential safety concerns. Additionally, the model’s SHAP-based interpretability allows pharmacovigilance experts to examine the specific linguistic features (e.g., drug names, symptom mentions) driving each prediction, supporting targeted manual review and causality assessment. By enabling prioritization of cases and focusing expert attention on high-risk signals, this framework enhances the efficiency and responsiveness of pharmacovigilance operations, ultimately contributing to improved patient safety and public health outcomes.

### Comparison with other parameter-efficient fine-tuning methods

To provide a comprehensive evaluation of our choice of LoRA for Adverse Drug Reaction (ADR) detection, we compare LoRA with other prominent parameter-efficient fine-tuning (PEFT) methods, including AdapterFusion, Prompt Tuning, BitFit, and QLoRA. This comparison addresses the trade-offs in computational efficiency, model performance, and compatibility with our dataset and transformer models (BERT, DistilBERT, RoBERTa), as well as their suitability for real-time pharmacovigilance applications.

#### Justification for selecting LoRA

LoRA was selected as the primary PEFT method due to its proven parameter efficiency, simplicity, and robust performance across various model sizes [22]. By employing low-rank matrix decomposition (δW = W__down_ * W__up_), LoRA updates only a small subset of parameters, ranging from 0.01% to 0.5% of the total parameters, thereby significantly reducing memory and computational costs. In our experiments (Table 3), LoRA reduced training time by approximately 50% (e.g., from 746.76 s to 378.13 s for RoBERTa), while maintaining classification accuracy above 98%. Furthermore, LoRA introduces no inference overhead [31], making it ideal for real-time pharmacovigilance systems where low latency is critical.

##### Comparison with other PEFT methods

**AdapterFusion **[32]**:** AdapterFusion enhances adapters by learning task-specific combinations of multiple adapter layers, improving performance in multi-task settings. However, it introduces additional parameters, accounting for 0.1–6% of the total parameters, and requires two forward passes for consistency regularization, thereby increasing memory usage during training [31]. For our small and noisy ADR dataset (3,937 samples), AdapterFusion’s increased complexity outweighs its benefits, as LoRA’s simpler architecture achieves comparable accuracy (e.g., 0.983 for DistilBERT, Table 3) with a lower memory footprint (433.43 MB for BERT with LoRA vs. 1258.57 MB for full fine-tuning).

**Prompt Tuning **[33]**:** Prompt Tuning prepends trainable soft prompts to input embeddings, achieving high parameter efficiency, with only 0.1% of the parameters [31]. However, it introduces inference overhead due to the increased sequence length, which scales quadratically with the transformer’s complexity [31]. This overhead is impractical for our real-time ADR detection needs. Additionally, Prompt Tuning’s performance is susceptible to model size, becoming comparable to full fine-tuning only at scales above 10B parameters [31], far exceeding the sizes of our models (up to 124 M parameters for RoBERTa). LoRA’s direct parameter updates offer better stability and efficiency for our dataset.

**BitFit **[34]**:** BitFit fine-tunes only bias terms 0.05–0.1% of parameters [31], providing extreme parameter efficiency. However, it underperforms in larger models (> 1B parameters) and is less effective in architectures like LLaMA, which lack bias terms [31]. For our study, where DistilBERT has fewer bias terms and high accuracy is critical for medical applications, BitFit’s limited capacity to capture complex patterns makes it less suitable than LoRA, which achieves superior performance (e.g., 0.983 accuracy for DistilBERT, as shown in Table 3).

**QLoRA **[23] **We incorporated** QLoRA for BERT and RoBERTa, leveraging 4-bit quantization to reduce memory usage further (e.g., 149.33 MB for BERT with QLoRA vs. 433.43 MB for BERT with LoRA, Table 3). QLoRA maintains comparable accuracy (0.9813 for BERT) but increases training time due to quantization overhead (1088.30 s for BERT with QLoRA vs. 368.50 s with LoRA). However, QLoRA is incompatible with DistilBERT due to its reduced architecture, which lacks standard attention layers with distinct query, key, and value projections, and the incompatibility of quantization libraries like bitsandbytes with DistilBERT’s weight matrices [23, 31]. This limitation aligns with our findings, as DistilBERT’s simplified design hinders the application of advanced quantization techniques.

##### Integration with SHAP for interpretability

Unlike AdapterFusion and Prompt Tuning, which introduce additional computational layers that may complicate interpretability, LoRA and QLoRA maintain model transparency by directly modifying weight matrices. This aligns well with SHAP’s feature attribution framework, as evidenced by our SHAP analysis (Fig. 5), which highlights key features, such as drug names (e.g., “rituxan”), in ADR predictions. This transparency is critical for clinical trust and regulatory compliance in pharmacovigilance applications [31].

LoRA and QLoRA were prioritized for their balance of computational efficiency, performance, and compatibility with our dataset and models. While AdapterFusion, Prompt Tuning, and BitFit offer viable alternatives, their increased complexity, inference overhead, or limited applicability make them less suitable for our resource-constrained, real-time ADR detection framework. Future work will explore AdapterFusion for multi-task scenarios, Prompt Tuning for larger models, and BitFit for bias-rich architectures, as suggested by Lialin et al. [31].

### Challenges of noisy and informal language in social-media pharmacovigilance

The broader discussion of this study highlights the multidimensional challenges inherent in applying large language models to social-media-based pharmacovigilance. Unlike structured biomedical corpora, Twitter and similar platforms present significant variability in data distribution, linguistic form, and contextual reliability. Consequently, even high-performing transformer-based architectures such as LoRA and QLoRA must contend with both quantitative limitations including dataset imbalance and scarcity and qualitative noise arising from the informal and dynamic nature of user-generated texts. The following subsections examine these two critical sources of uncertainty class imbalance and linguistic noisiness and conclude with overarching methodological reflections and recommendations for future research.

#### Class imbalance considerations

A key limitation of this study lies in the inherent class imbalance across ADR categories, particularly the dominance of diabetes-related tweets compared to the underrepresented High_BP class. Such imbalance can skew the model’s decision boundary toward the majority class, increasing the probability of false negatives in minority categories. To mitigate this bias, we adopted a stratified data split ensuring proportional label distribution across training, validation, and test subsets. Furthermore, a class-weighted cross-entropy loss was employed to penalize misclassifications of rare classes more heavily. Performance evaluation emphasized macro-averaged F1-scores and confusion matrix analysis, which provide a balanced assessment across all classes and reveal the asymmetry in recall between frequent and infrequent ADR mentions.

SHAP-based interpretability analysis further demonstrated that the model retained semantically and clinically coherent attention patterns even for minority classes, suggesting that it did not merely memorize high-frequency linguistic tokens but captured relevant biomedical concepts such as drug–symptom associations. Nevertheless, the residual imbalance likely limits generalizability under highly skewed real-world pharmacovigilance scenarios. In future work, we plan to incorporate advanced imbalance-handling strategies such as synthetic oversampling (SMOTE), data augmentation using paraphrase-based transformers, and dynamic class weighting during fine-tuning to enhance robustness across rare ADR subpopulations.

#### Noisy and informal language in social media texts

Another inherent challenge in social-media-based pharmacovigilance arises from the noisy and informal linguistic characteristics of Twitter data, including the use of slang, abbreviations, misspellings, and hashtags. Although a multi-stage preprocessing pipeline comprising token normalization, hashtag decomposition, lemmatization, and abbreviation expansion was implemented to mitigate lexical variability, semantic drift and pragmatic ambiguity remain non-trivial. In particular, sarcasm and figurative expressions (e.g., “This pill almost killed me ) can mislead the model into assigning false ADR labels, as the literal sentiment diverges from the intended meaning.

To address these limitations, contextualized language models (e.g., BERT, RoBERTa) were deliberately employed due to their bidirectional encoding of contextual semantics, enabling partial disambiguation of informal and sarcastic content. Moreover, the SHAP interpretability analysis confirmed that the model tends to focus on clinically relevant tokens (e.g., drug names, symptom terms) rather than stylistic or emotional markers. Nevertheless, noise remains an inevitable property of user-generated content. Future extensions may incorporate sarcasm detection submodules, context-aware sentiment calibration, or cross-platform textual fusion (e.g., combining Reddit and patient forums) to improve contextual robustness in pharmacovigilance tasks.Despite these linguistic challenges, the proposed framework maintains interpretability, which offers an avenue to better understand model reasoning and contextual grounding.

#### Limitations and future work

The primary objective of this study was to develop a computationally efficient and scientifically interpretable framework for adverse drug reaction (ADR) detection using parameter-efficient adaptation of large language models. Within this scope, the framework successfully demonstrates that low-rank fine-tuning through LoRA and QLoRA can significantly reduce computational overhead while maintaining high predictive performance and transparent, clinically meaningful interpretability through SHAP analysis. Nonetheless, several considerations lie beyond the present study’s immediate scope but represent important directions for future advancement.

First, the analysis was intentionally conducted on a relatively small, English-language Twitter dataset comprising approximately 3,937 annotated ADR-related posts. This design choice reflects the study’s focus on modeling under real-world, high-noise, low-resource conditions typical of user-generated social media data where linguistic informality and semantic ambiguity present major challenges. Although this setup provides a rigorous environment to assess the model’s robustness and interpretability, it naturally limits cross-lingual and cross-platform generalizability. Future extensions will therefore explore multilingual and multi-source pharmacovigilance, integrating textual data from diverse platforms such as Reddit, Facebook, and patient forums, as well as from formal biomedical repositories, to enhance contextual and cultural adaptability.

Second, while the proposed model effectively captures semantic and clinical correlations within unstructured patient discourse, it has not yet been adapted to structured biomedical corpora such as clinical narratives, EHRs, or regulatory reports. Extending the framework toward cross-domain pharmacovigilance through domain adaptation, transfer learning, and ontology-guided fine-tuning will allow it to bridge informal patient-reported data with clinically validated pharmacovigilance knowledge sources, thereby improving medical reliability and interoperability across heterogeneous text environments.

Third, although SHAP-based interpretability provides transparent, feature-level explanations of model behavior, true clinical validation requires expert assessment. The current study did not include a human-in-the-loop validation phase, which remains an essential step toward ensuring biomedical causality and regulatory trustworthiness. Incorporating iterative expert feedback in future iterations will align algorithmic reasoning with clinical expertise and support compliance with oversight requirements of agencies such as EMA and FDA.Furthermore, achieving regulatory-grade interpretability will require extending the current SHAP-based framework toward causal explanation and traceable decision reasoning, supported by expert-in-the-loop validation and knowledge-graph integration to ensure compliance with emerging EMA and FDA guidelines.Finally, from a deployment perspective, this framework was designed primarily for computational efficiency and interpretability, not for real-time processing at Twitter scale. However, the architecture provides a strong foundation for future scalability. Emerging narrative-based interpretability methods in large language models (LLMs) which generate concise natural-language explanations rather than computationally intensive token-level attributions—offer a viable path toward real-time pharmacovigilance monitoring. When combined with retrieval-augmented generation (RAG) or domain-aware summarization, such approaches could enable scalable, low-latency, and contextually grounded analysis across multilingual and multi-platform data streams.

In summary, while this study defines and validates a lightweight, interpretable, and computationally optimized framework for ADR detection in social-media data, future research will expand its scope toward multilingual, cross-domain, expert-validated, and real-time pharmacovigilance systems. Such evolution will transform the current prototype into a scalable, clinically reliable, and globally applicable pharmacovigilance intelligence framework.

## Conclusion

This study demonstrates that integrating parameter-efficient fine-tuning techniques, such as LoRA and QLoRA, with transformer-based models enables the accurate, interpretable, and resource-efficient detection of adverse drug reactions from noisy, real-world social media data. By combining LoRA/QLoRA with SHAP-based explainability, our framework maintains high predictive performance across multiple disease classes while reducing memory and computational requirements by up to 50%, paving the way for scalable, real-time pharmacovigilance applications on hardware-limited platforms. Our systematic comparison highlights the unique balance of transparency, efficiency, and robustness achieved by this approach, which is particularly valuable for real-time health monitoring and decision support in dynamic, data-rich environments. Future research should explore the extension of this framework to multilingual datasets, additional social media platforms, and larger-scale deployment. Further benchmarking against emerging parameter-efficient fine-tuning methods and incorporating domain-specific knowledge sources may further enhance accuracy and generalizability. Collectively, our findings underscore the practical feasibility and clinical relevance of adopting efficient and interpretable deep learning solutions for next-generation pharmacovigilance.

## Figures and Tables

**Fig. 8:**
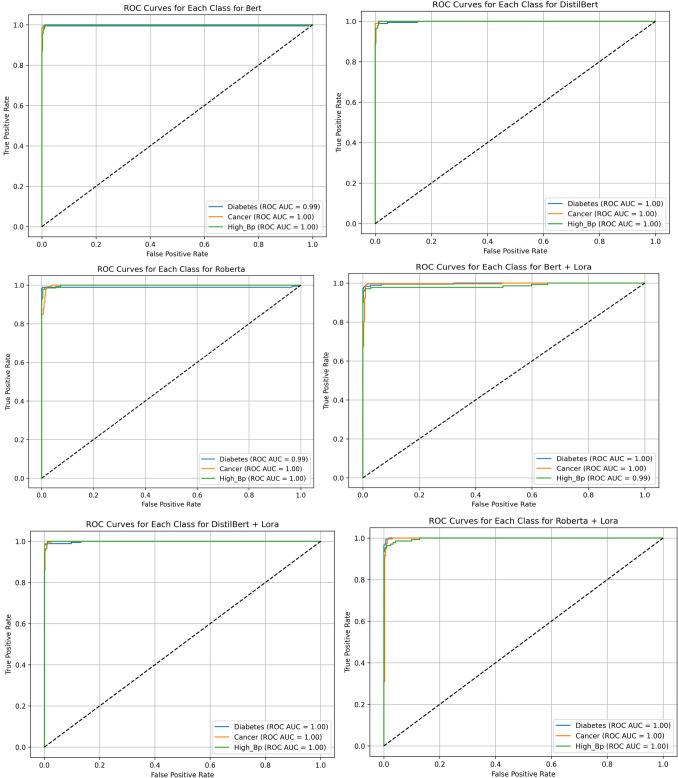

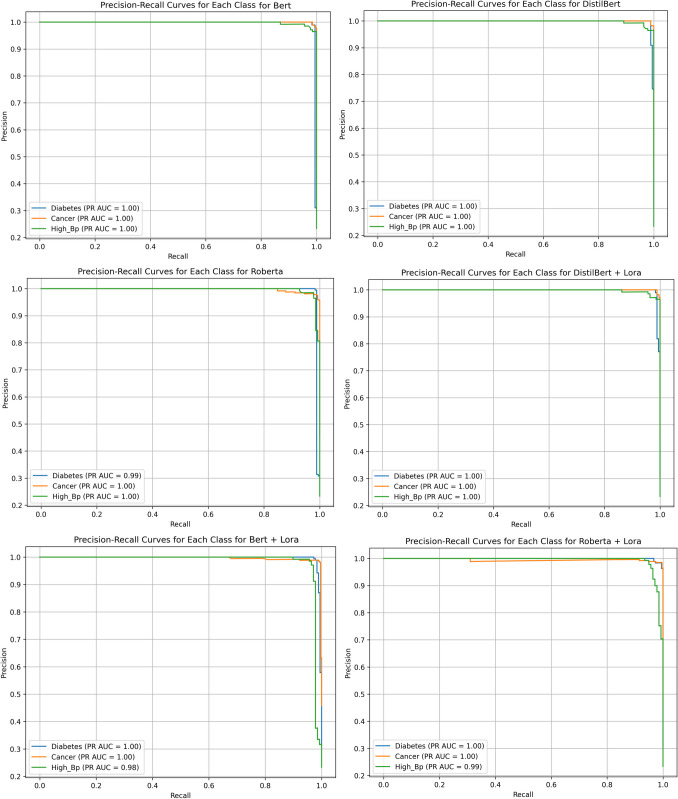

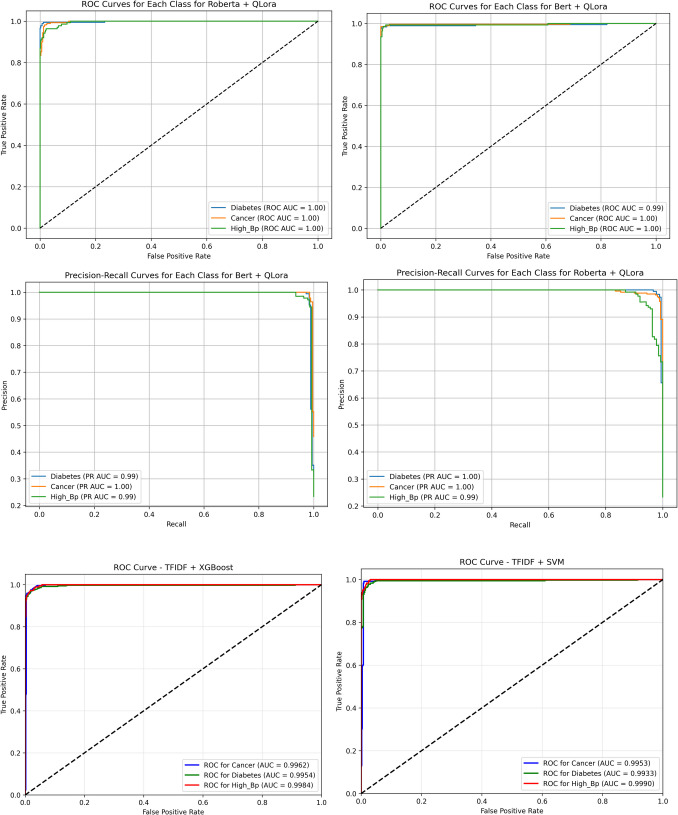
Class-wise One-vs-Rest ROC Curves and Micro/Macro-Averaged AUCs for ADR Detection Across Models and Fine-Tuning Strategies

## Data Availability

The codes and dataset of the proposed method are available on GitHub:https://github.com/Datargets/ADR_XAI

## Appendix

The Appendix extends the core evaluation by presenting one-vs-rest ROC curves for each disease class, along with micro- and macro-averaged AUCs across all model–fine-tuning combinations. These visualizations provide a comprehensive view of sensitivity–specificity trade-offs beyond single-threshold performance metrics.

One-vs-rest ROC curves for each disease class (Diabetes, Cancer, High_BP) together with micro- and macro-averaged AUCs across all models (BERT, DistilBERT, RoBERTa) and fine-tuning regimes (Full, LoRA, QLoRA). These plots complement the main text’s accuracy/MCC tables by characterizing the full operating range of sensitivity–specificity trade-offs, rather than a single threshold outcome. Consistent with the headline metrics (≥ 97% accuracy across settings), the ROC envelopes for the best models cluster near the top-left region, indicating strong separability of ADR mentions from non-targets across classes. In other words, the Fig. 8 curves explicitly show that the high end-to-end performance in Table 3 stems from the robust ranking of class probabilities, rather than merely favorable thresholding.

Class-wise ROC shapes highlight an expected asymmetry across labels. Diabetes and Cancer curves are closest to the ideal corner, reflecting the models’ ability to exploit clear lexical anchors (drug names and salient symptom mentions). By contrast, High_BP exhibits a modest rightward shift at low false-positive rates, aligning with the confusion-matrix analysis in the main text, which revealed comparatively higher false negatives for this class under noisy, informal language. This behavior is consistent with the class imbalance and greater linguistic ambiguity in High_BP tweets; it also aligns with the SHAP evidence that clinically specific tokens (e.g., “Rituxan,” “Lantus,” “Losartan,” and symptom terms) drive confident decisions. In contrast, generic function words contribute little to discrimination. Together, these diagnostics explain why class-wise AUC is strongest where ADR cues are most explicit and slightly attenuated where signals are sparser or noisier. Across parameter-efficient regimes, the ROC–AUC profiles of LoRA variants closely track their full-fine-tuned baselines, indicating that the significant gains in efficiency do not come at the expense of ranking quality. This mirrors the small deltas observed in accuracy and MCC (e.g., BERT Full vs. BERT-LoRA/QLoRA), while delivering over 100 × reductions in trainable parameters and substantial memory savings. QLoRA further reduces memory with a training-time trade-off. In the ROC view, this manifests as tightly overlapping curves for Full and LoRA (and, where applicable, QLoRA), with macro-AUCs remaining within a narrow band across backbones. These plots therefore reinforce the research’s central claim: LoRA preserves discriminative capacity while materially improving computational efficiency, supporting deployment in constrained environments.

From a practical perspective, the ROC–AUC visualizations help select operating points tailored to pharmacovigilance risk tolerance. For surveillance pipelines that prioritize low false-positive rates (to limit unnecessary human review), the curves show that LoRA models sustain high true-positive rates in clinically relevant regions. Conversely, when early-signal sensitivity is paramount, modest threshold shifts increase recall with minimal loss of specificity. Combined with the class-wise error patterns (confusion matrices) and token-level explanations (SHAP), Fig. 8 AUCs provide a convergent picture: parameter-efficient models rank examples correctly across a wide score range and do so for clinically interpretable reasons, making them suitable candidates for real-time ADR monitoring at scale. Overall, results confirm that the proposed framework sustains high discriminative power across diverse classes while delivering substantial efficiency gains. The close alignment between Full and LoRA ROC profiles demonstrates that parameter-efficient tuning can meet the dual demands of accuracy and scalability. These findings strengthen the case for LoRA-based ADR detection as a viable solution for operational pharmacovigilance in resource-constrained settings.

## Footnotes

1 https://github.com/Datargets/ADR_XAI

2 https://github.com/sethitanvi/Adverse-Drug-Reaction
